# Investigating Amphoteric 3,4′-Biscoumarin-Based *ortho*-[(Dialkylamino)methyl]phenols as Dual MAO and ChE Inhibitors

**DOI:** 10.3390/ijms262010197

**Published:** 2025-10-20

**Authors:** Anthi Petrou, Caterina Deruvo, Rosa Purgatorio, Boris Lichitsky, Andrey N. Komogortsev, Victor G. Kartsev, Modesto de Candia, Marco Catto, Cosimo D. Altomare, Athina Geronikaki

**Affiliations:** 1 Department of Pharmaceutical Chemistry, School of Pharmacy, Aristotle University of Thessaloniki, 54124 Thessaloniki, Greece; anthi.petrou.thessalonik1@gmail.com; 2 Department of Pharmacy-Pharmaceutical Sciences, University of Bari Aldo Moro, Via E. Orabona 4, 70125 Bari, Italy; caterina.deruvo@uniba.it (C.D.); rosa.purgatorio@uniba.it (R.P.); modesto.decandia@uniba.it (M.d.C.); marco.catto@uniba.it (M.C.); 3 Zelinsky Institute of Organic Chemistry, Leninsky Prospect, 119991 Moscow, Russia; blich2006@mail.ru (B.L.); dna5@mail.ru (A.N.K.); 4 InterBioScreen Ltd., 119019 Moscow, Russia; vkartsev@ibscreen.chg.ru

**Keywords:** biscoumarin, monoamine oxidases, cholinesterases, inhibitors, molecular docking, ADME profile, Alzheimer’s disease

## Abstract

Nineteen previously and newly synthesized amphoteric 8-[(dialkylamino)methyl]-7-hydroxy-4-(2-oxo-2*H*-chromen-3-yl)-2*H*-chromen-2-ones were assayed as inhibitors of monoamine oxidases (MAO-A and B) and cholinesterases (AChE and BChE). Five of the tested compounds (**2b**, **2c**, **3c**, **5b**, and **5c**), namely those bearing the less bulky alkyls in the Mannich base 8-CH_2_NR_2_ (R = Me, Et) and the halogens (Cl, Br) at C6 of the 4-coumarin-3-yl moiety, showed moderate inhibitory potencies toward human MAO-A in the single-digit micromolar range (IC_50_s from 1.49 to 3.04 µM). In particular, the 6′-Cl derivatives **2b** and **5b** proved to be reversible competitive inhibitors of human MAO-A with *K*_i_ values of 0.272 and 0.326 µM. Among the tested compounds, **3c** proved to also be a moderate inhibitor of human AChE (IC_50_ 4.27 µM). Molecular docking calculations suggested binding modes of the most active compounds to MAO-A and AChE binding sites consistent enough with the experimental data. Chemoinformatic tools suggest for the most active compounds, including the dual MAO-A/AChE inhibitor **3c**, full compliance with Lipinski’s rule of five, high probability of gastrointestinal absorption, but low blood–brain barrier (BBB) permeability. While further efforts are required to improve their CNS distribution, herein new phenolic Mannich bases have been identified that may have potential for treating neurodegenerative syndromes.

## 1. Introduction

Alzheimer’s disease (AD) is the leading neurodegenerative disease, with a strong impact on health and quality of life. People suffering from AD have learning deficits along with an impairment of memory and language skills due to the disruption of cholinergic transmission in hippocampal areas [1]. As a result of the so-called ‘cholinergic hypothesis’, up to date, the pharmacological therapy of AD has essentially been based on the restoration of adequate levels of acetylcholine (ACh) ensured by acetylcholinesterase (AChE) inhibitors (i.e., rivastigmine, galantamine, and donepezil) [2], but also butyrylcholinesterase (BChE) inhibitors. Memantine, an antagonist of the NMDA (*N*-methyl-d-aspartate) receptor, is also used in the therapy of moderate to severe forms of AD [3]. Nevertheless, all the mentioned drugs do not cure the disease but exert only symptomatic relief. Therefore, the need for new drugs against AD remains a challenging task. Monoclonal antibodies, such as aducanumab, lecanemab, and donanemab [4], deserve great hope as disease-resolving therapies, but approved therapies have so far shown contrasting results in terms of efficacy and harmful side effects.

In the last two decades, the multitarget approach to drug design has been considered as a promising strategy in discovering neurotherapeutics against AD [5], paying attention to other related mechanisms of neurotoxicity. In particular, oxidative stress has been considered an interplaying event with the deposition of amyloid plaques and neurofibrillary tangles [6]. In turn, deregulation of endogenous redox systems and over-production of radical species lead to lipid peroxidation and nucleic acid mutations. In this context, a neuroprotective activity against oxidative stress has been claimed for inhibitors of monoamine oxidases. The flavoenzyme monoamine oxidase (MAO, EC 1.4.3.4), which catalyzes the oxidative deamination of dietary and neurotransmitter amines, including serotonin, tryptamine, dopamine, norepinephrine, epinephrine, β-phenylethylamine, tyramine, and octopamine, has been considered as an anti-AD drug target. MAO exists in two isoforms, MAO-A and MAO-B, sharing about 70% sequence identity and showing distinct substrate and inhibitor specificities [7]. MAO-A preferentially metabolizes serotonin and norepinephrine, while MAO-B preferentially metabolizes phenylethylamine, benzylamine, and dopamine.

According to their selectivity, MAO subtypes are involved in various neurodegenerative pathologies, including AD, Parkinson’s disease (PD), and depression. Age-related increase in MAO expression levels is a significant causative factor in the etiology of neurodegenerative illnesses and mental disorders. Selective MAO-A inhibitors (MAOAIs) find use in the treatment of depression, whereas MAOBIs are used in alternation to levodopa to treat PD. First-generation MAO inhibitors, such as iproniazid, phenelzine, and tranylcypromine, are not isoform-selective and their use was associated with severe side effects [8]. Currently, selective MAOAIs (moclobemide) and MAOBIs (l-deprenyl and safinamide) are preferred because of their better therapeutic index, but with limited prevalence compared with safer and more effective drugs. Recently, a possible repositioning of MAOs as a target in chemoresistant tumors has boosted a renewed attention in MAO inhibitors, particularly for MAOAIs that have demonstrated synergistic efficacy in the antitumor therapy of recurrent prostate cancer, metastatic breast cancer, and glioma [9].

**Figure 1:**
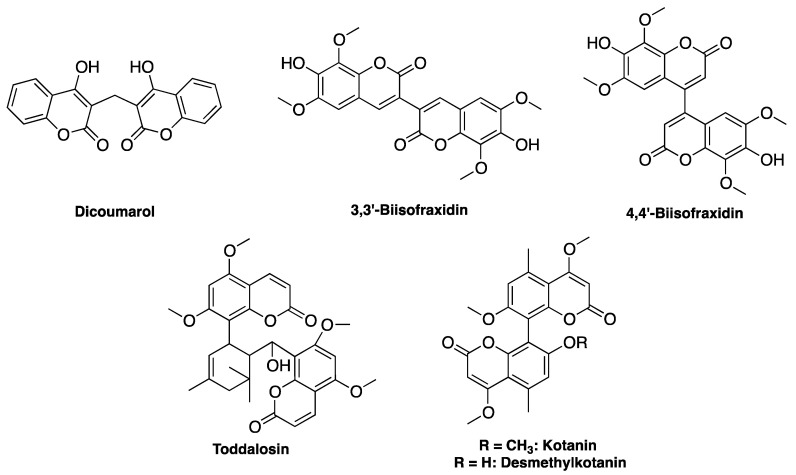
Representative structures of biologically active biscoumarin derivatives.

Among the numerous MAOIs developed, coumarin-based derivatives are well-documented MAO inhibitors with good MAO-A/B selectivity ratios, depending upon the nature and pattern of their substitution, and great potential as multitarget agents for neurodegenerative diseases [10]. Coumarins have a high electron count and exhibit good charge transport capabilities [11]. The coumarin moiety taken as a scaffold is synthetically versatile and widely used in the preparation of biologically active molecules. Indeed, coumarin derivatives have found a wide variety of applications for their anticoagulant, anti-inflammatory, anticancer, antidiabetic, anti-neurodegenerative, antioxidant, antidepressive, and antimicrobial effects [12]. Coumarin dimers (i.e., biscoumarins), mostly of natural origin, also form a bioactive scaffold. Very often these are symmetrical homodimers, like, for example, dicoumarol and its substituted derivatives [13,14], 3,3′-biisofraxidin (from *Sarcandrae Herba*) [15] and 4,4′-biisofraxidin (from *Impatiens balsamina*) [16], 8,8′-biscoumarins like toddalosin (from *Toddalia asiatica*) [17], and the mycotoxins kotanin and desmethylkotanin (from *Aspergillus glaucus*) [18] (Figure 1). Biscoumarins also attracted significant interest due to their wide range of biological activities such as antimicrobial [19], anti-inflammatory [20], anticancer [21], anti-obesity [22], antiviral [23,24], antidiabetic [25], MAO inhibitory [26], and anticholinesterase [27,28,29] activities.

**Scheme 1:**
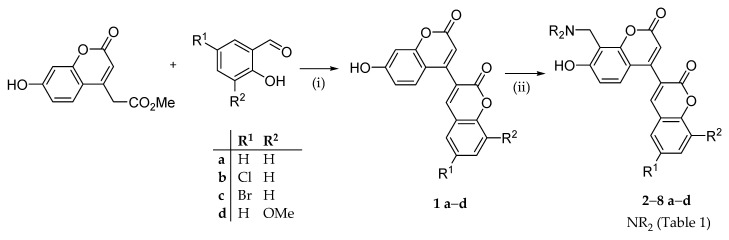
Synthesis scheme of 8-[(dialkylamino)methyl]-4-(2-oxo-2*H*-chromen-3-yl)-7-hydroxy-2*H*-chromen-2-one derivatives. Reagents and conditions: (i) DBU, abs. 1,4-dioxane (DMF for **1d**), 100–105 °C, 4–10 h; (ii) suitable aminal, 1,4-dioxane, reflux, 3–5 h.

Several years ago, Frasinyuk et al. reported the aminomethylation of 7-hydroxy-4-(2-oxo-2*H*-chromen-3-yl)-2*H*-chromen-2-one (Scheme 1) [30], synthesizing diverse amphoteric *ortho*-phenolic Mannich bases (i.e., 8-[(dialkylamino)methyl]-7-hydroxy substituents) on the 2*H*,2′*H*-3,4′-bichromene-2,2′-dione (hereinafter referred to as 3,4′-biscoumarin) scaffold. The online PASS (prediction of activity spectra for substances) software (https://way2drug.com/PassOnline/, accessed on 20 June 2025) [31] predicted for forty diverse amphoteric 3,4′-biscoumarin derivatives indicated above MAO inhibitory activity with a Pa value (i.e., the probability of a compound to be active) in the range 0.203–0.895. Note that most of the compounds had Pa values lower than 0.5, indicating on one hand their relative novelty compared to the molecular structures in the PASS training set and on the other hand, the low probability of being active as MAOIs.

Due to our interest toward novel coumarin cores as a scaffold of novel MAOIs, endowed with further anti-AD activities, such as ChE inhibition, herein we screened nineteen previously and newly synthesized 3,4′-biscoumarins bearing the amphoteric 8-[(dialkylamino)methyl)-7-hydroxy group as dual MAO/ChE inhibitors, along with docking calculation, solubility determination, and in silico drug-likeness.

## 2. Results and Discussion

### 2.1. Chemistry

Nine new 3,4′-biscoumarin-containing amphoteric derivatives (**2a**–**d**, **3c**, **4d**, **5d**, **6d**, and **8d**) were synthesized as previously described [30] according to Scheme 1. 7-hydroxy-3,4′-biscoumarins (**1a**–**d**) were prepared by the condensation of 7-hydroxy-coumarin-4-methylacetate with substituted salicylic aldehydes in dioxane (or DMF for **1d**) in the presence of DBU (1,8-diazabicyclo [5.4.0]undec-7-ene) as a catalyst. Regarding the aminomethylation procedure, the aminals of the secondary amines were refluxed in dioxane with a product yield between 48 and 82%. In this case, the electrophilic substitution took place exclusively at C8, as confirmed by the NMR spectra [30].

The structural exploration was primarily focused on the variation in terms of the hydrophobicity and steric effects of the alkyls of the aminomethyl group at C8 (CH_2_NR_2_) of the coumarin moiety. The congeners (**b**–**d**), bearing the halogens Cl or Br (as R^1^) at C6 and OMe (as R^2^) at C8 of the coumarin-3-yl moiety, available in our molecular library, were also investigated.

### 2.2. Structure–Activity Relationships

#### 2.2.1. In Vitro Inhibition of Monoamine Oxidases and Cholinesterases

All nineteen compounds were tested against each enzyme, i.e., human *hu*MAO-A and *hu*MAO-B isoforms, electric eel *ee*AChE, and horse serum *eq*BChE, at 10 μM concentration, and for those exhibiting more than 60% inhibition at that concentration, the IC_50_ values were determined. The inhibition of human *hu*AChE was also evaluated for five compounds. Clorgiline and donepezil were used as positive controls in the MAO and ChE inhibition assays, respectively. The enzymes’ inhibition data (expressed as IC_50_s or % inhibition at 10 µM concentration) are summarized in Table 1 as mean ± SD from three independent experiments.

**Figure 2:**
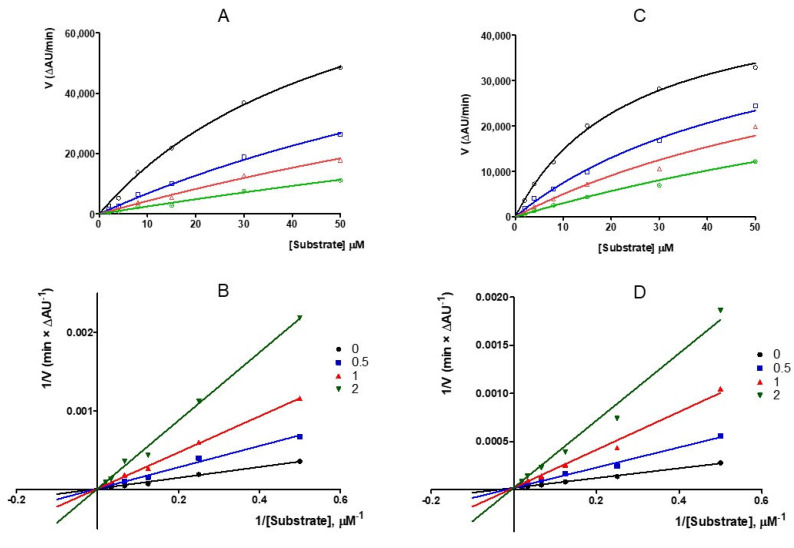
Michaelis–Menten (**A**,**C**) and Lineweaver–Burk (**B**,**D**) plots of inhibition kinetics of MAO-A for compounds **2b** (**A**,**B**) and **5b** (**C**,**D**).

For the sake of clarity, the position of the Mannich base (-CH_2_NR_2_) on the main coumarin core will be indicated as C8, whereas the positions of Cl or Br and OMe on the 4-coumarin-3-yl moiety will be indicated as C6′ and C8′. As can be seen in Table 1, about half of the screened compounds significantly inhibited the two MAO isoforms at 10 µM concentration, achieving a slight selectivity toward MAO-A. The most potent MAO-A inhibitors were **2b** (IC_50_ 1.49 µM) and **5b** (IC_50_ 1.85 µM) bearing NMe_2_ and N(Me)*n*Bu, respectively, as NR_2_ at C8 and Cl as R^1^ at C6′. The inhibition kinetics determined on MAO-A for the most potent inhibitors **2b** and **5b** assessed a competitive mode of inhibition, with no variation in *v*_max_ upon increasing the inhibitors’ concentration (Figure 2). The inhibition constant (*K*_i_) values for **2b** and **5b** resulted in 0.272 ± 0.043 and 0.326 ± 0.035 µM, respectively.

A time course of the inhibition kinetics, measured spectrophotometrically as the increase in absorbance at 316 nm of 4-hydroxyquinoline, i.e., the product of MAO-mediated oxidation of kynuramine as a substrate, confirmed the reversibility of enzyme inhibitor binding (Figure S11, Supplementary Materials).

Three further compounds, namely the 6′-Br congeners **2c**, **3c**, and **5c**, proved to act as MAO-A inhibitors with single-digit micromolar IC_50_s. The inhibition potency appeared to depend on the hydrophobicity and steric hindrance of the aminomethyl group at C8. Within the limits of the explored property space, the data suggest that the anti-MAO (preferentially anti-MAO-A) activity is primarily affected by the size of the tertiary amine of the Mannich base at C8. As a trend, tertiary amines with a double-branched alkyl group (subset **7**) or larger than Et (subsets **4**, **6**, and **8**) are less tolerated than NMe_2_ (**2**), NEt_2_ (**3**), and N(Me)*n*Bu (**5**). The halogens at C6′ appear to enhance MAO-A inhibition potency, with Cl exerting a slightly stronger effect than Br. In contrast, the introduction of a methoxy group at C8′ decreases the inhibitory activity against MAO-A.

Regarding the cholinesterase inhibition, most of the screened derivatives proved to preferentially inhibit *ee*AChE over *eq*BChE. Only four compounds (**3c**, **4d**, **5c**, and **5d**) achieved finite IC_50_ values at concentrations less than 10 μM. The most potent *ee*AChE inhibitor was **3c** (IC_50_ 1.56 μM), which also exhibited MAO-A inhibition at a very close IC_50_ (3.04 μM). The optimal AChE inhibition potency was achieved for -CH_2_NEt_2_ and -CH_2_N*n*Pr_2_ at C8, with 6′-Br (**3c** and **5c**) or 8′-OMe (**4d** and **5d**) exerting favorable effects. Compounds **2b**, **2c**, **3c**, **4d**, and **5b**, tested as *hu*AChE inhibitors, confirmed the activity trend observed with *ee*AChE. Interestingly, the 8′-OMe congener **4d** achieved an IC_50_ value of 0.97 µM.

Overall, in the series examined, **3c** appears to be the one that inhibits both MAO-A and AChE with a single-digit micromolar IC_50_ and a slight selectivity over MAO-B and BChE, respectively.

#### 2.2.2. Molecular Docking Calculations

To shed light on the molecular factors mainly responsible for the more efficient binding modes and higher inhibition potencies of the examined amphoteric biscoumarins to the target enzymes, molecular docking calculations were carried out using AutoDock software (release 4.2). The main results obtained from the docking simulation of most of the compounds with a crystal structure of human MAO-A, in complex with clorgiline (pdb 2BXR), or human MAO-B (pdb 1GOS) are reported in the Supplementary Materials (Table S1), whereas in Table 2, the main docking calculation results are summarized, which pertain to the highest-scored docking poses to MAO-A of **2a**, **2b**, **2d**, **4a**, **5a**, and **5b**, which can help in understanding the key factors modulating the inhibition potency of the compounds under investigation.

**Figure 3:**
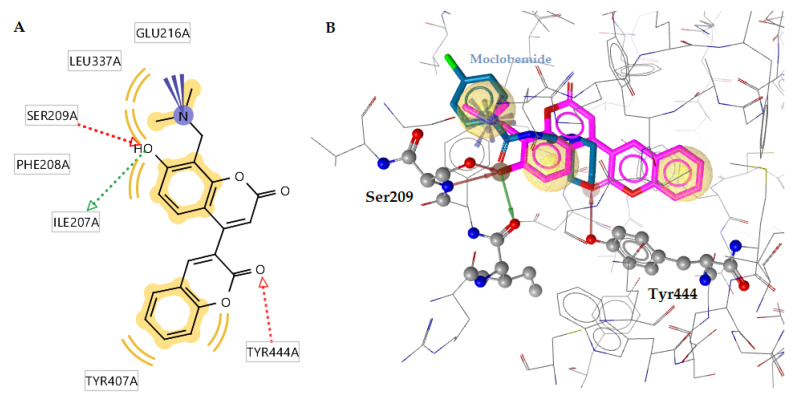
(**A**) Two-dimensional diagram of the lead compound **2a** into the MAO-A binding site. (**B**) Superposition of the structure of **2a** (pink) and moclobemide (blue), a well-known selective MAO-A inhibitor, into the active site of the MAO-A enzyme. For clarity, he H atoms have been removed. Red (donor) and green (acceptor) dotted or solid arrows indicate H-bond interactions, and yellow curved lines (**A**) or spheres (**B**) hydrophobic interactions.

**Figure 4:**
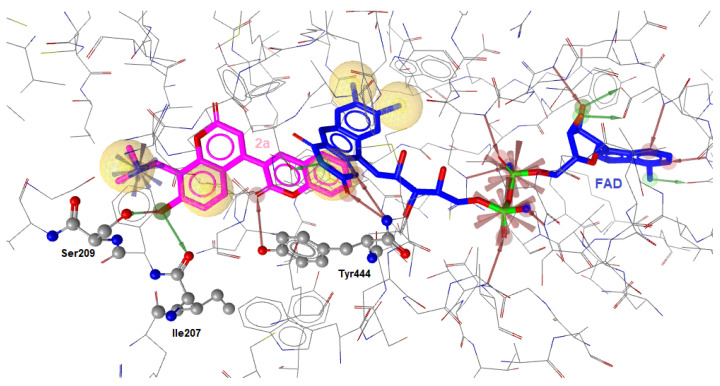
The highest-scored docking pose of the biscoumarin compound **2a** (pink) and FAD (blue) into the active site of the MAO-A enzyme. For clarity, the H atoms have been removed. Red (donor) and green (acceptor) solid arrows indicate H-bond interactions, and yellow spheres hydrophobic interactions.

In Table 2, besides the free energy of binding (FEB, kcal·mol^−1^), the residues involved in H-bonding, and hydrophobic/aromatic and ionic interactions with the ligand are reported. To aid in the interpretation of the potency ranking, the residues that were established as playing a role in substrate and inhibitor selectivity are highlighted in bold [32]. The 2D graphical representations of the best-scored docking pose of the lead **2a** (NR_2_ = NMe_2_, Figure 3), compared to those of the less active **4a** (NR_2_ = N(*n*Pr)_2_, Figure 4), could suggest why and how the different MAO-A inhibitory potency may relate to the diverse binding mode.

Unlike MAO-B, whose FAD binding site has two distinct cavities, i.e., the entrance and substrate cavities separated by a gate (Tyr326), MAO-A has one single hydrophobic cavity (about 550 Å^3^ volume), in which Phe208 and Ile335 play significant roles in substrate and inhibitor selectivity, whereas Tyr407 and Tyr444 may favor ligand binding through aromatic stacking or H-bond formation through their phenolic OH groups [33].

By orienting the coumarin moiety into a flexible cavity primarily lined by Phe208 and Val210 through hydrophobic contacts, the biscoumarin **2a** may bind MAO-A strongly. The coumarin benzene ring is well accommodated into the catalytic cleft facing the FAD cofactor (Figure 4) by the formation of stable hydrogen bonding through Tyr444 (1.59 Å). Two residues, namely Ser209 and Ile207, should contribute to stabilizing the complex interacting with the phenolic OH at C7 of the inhibitor as the H-bond donor (2.87 Å) and acceptor (3.05 Å), respectively. Moreover, the protonated tertiary amine of **2a** may attain a salt bridge strengthened by H-bonds with Glu216. In addition, several hydrophobic residues, such as Phe208, Val303, Ile335, Phe352, Tyr444, and Met445, should form extra hydrophobic interactions with the inhibitor into the active site (Figure 3A). The drug moclobemide, taken as a reference MAOAI, exhibited a similar binding mode (Figure 3B). Figure 3 highlights an almost parallel aromatic stacking between flavin and distal coumarin moiety.

**Figure 5:**
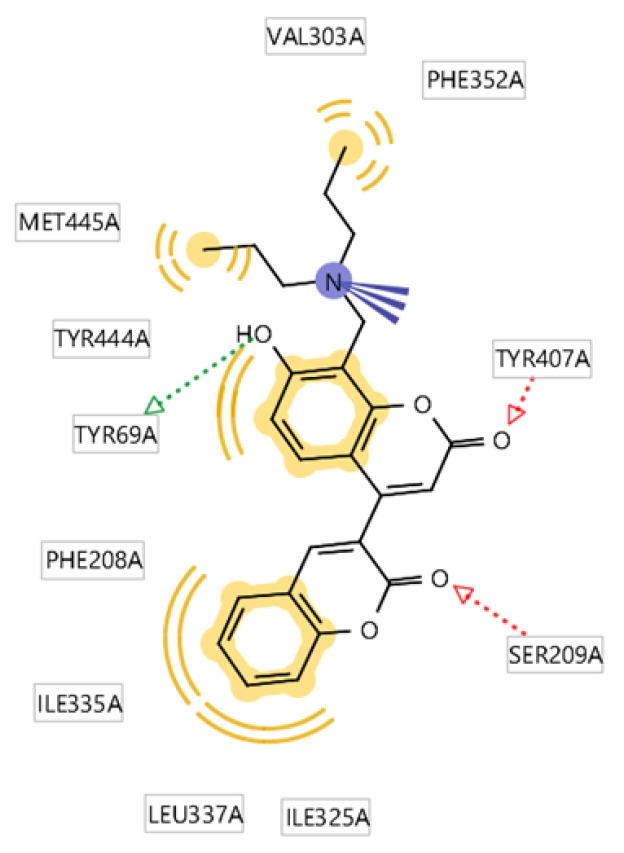
Two-dimensional diagram of compound **4a** into the binding site of MAO-A. Red (donor) and green (acceptor) dotted arrows indicate H-bonds, and yellow shadow curved lines hydrophobic interactions.

With the increasing size of the alkyls on the tertiary amino groups beyond the ethyl groups, like in **4a** (Figure 5), it can be reasonably hypothesized that the potency decrease could be due to a change in the binding mode with a decrease in the ligand efficiency. Indeed, the interactions of the *n*-propyl groups of the tertiary amine in **4a** with the hydrophobic residues (Tyr444, Met445, Val 303, Phe352) most likely do not compensate the loss of electrostatic interactions and H-bonding achieved by the NMe_2_ group with Glu216. In addition to the hydrophobic and aromatic interactions around the two coumarin moieties, two key interactions characterize the different (less efficient) binding mode of **4a** compared to that of the more active **2a**, i.e., Ser209 as the H-bond donor to the carbonyl O of the second coumarin nucleus (instead of the phenolic OH) and Tyr407 as the H-bond donor to the O of the first coumarin nucleus.

**Figure 6:**
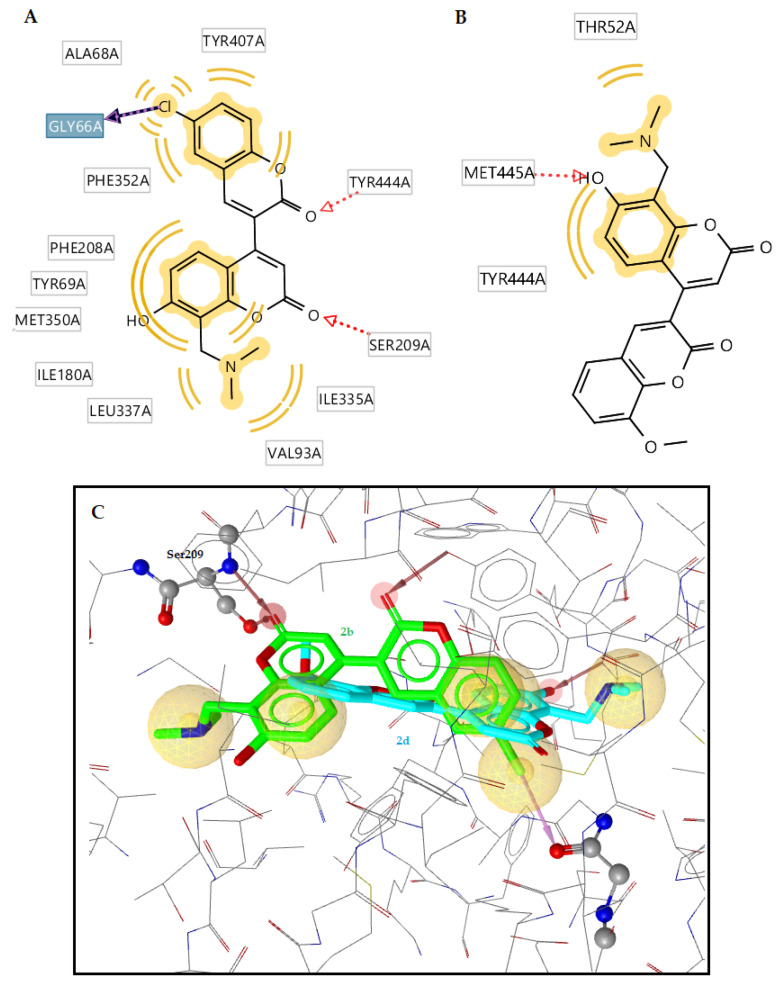
(**A**) Two-dimensional diagram of compound **2b** in the binding site of MAO-A. (**B**) Two-dimensional diagram of compound **2d** in MAO-A. (**C**) Superposition of **2b** (green) and **2d** (cyan) in the active site of MAO-A enzyme. Red dotted (**A**,**B**) or solid (**C**) arrows indicate H-bonds, purple halogen bonds, and yellow shadows (**A**,**B**) or spheres (**C**) hydrophobic interactions.

Compound **2b** (6′-Cl congener) achieved higher activity against MAO-A compared to the unsubstituted **2a** (Figure 6). Similarly to moclobemide, it forms two key H-bonds with active site residues Ser209 and Tyr444, which suggests a similar binding mode. Moreover, the presence of the 6′-Cl substituent appears to enhance binding by stabilizing the molecule within the enzyme’s active site, through a halogen bond interaction with Gly66, which might contribute to a more favorable orientation and interaction profile (Figure 6A). In contrast, compound **2d** (8′-OMe congener) shows a different orientation in the active site (Figure 6B) and a less efficient binding mode. The 8′-OMe group is directed toward the core of the binding pocket in an unfavorable conformation, disrupting optimal positioning and weakening key interactions (Figure 6C). This might likely explain its reduced activity compared to **2b**.

**Figure 7:**
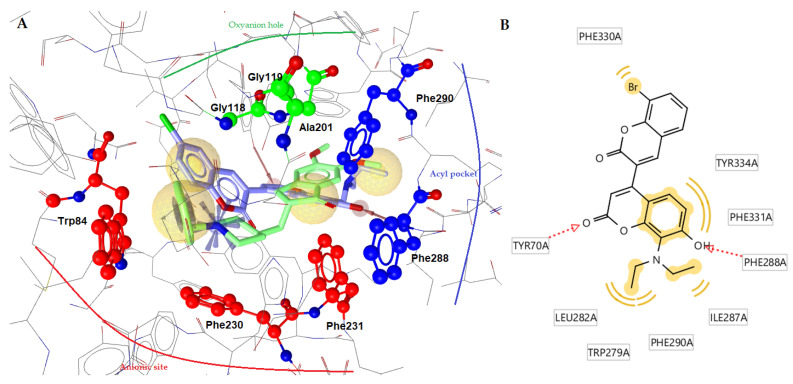
(**A**) Superposition of compound **3c** (purple) and donepezil (green) into the active site of AChE. (**B**) Two-dimensional diagram of compound **3c** into the AChE active site. The H atoms have been removed for the sake of clarity. The side chains of the interacting residues are represented as balls and sticks and diversely colored according to the enzyme subsite. Red solid (**A**) or dotted (**B**) arrows indicate H-bonds, and yellow spheres (**A**) or shadow curved lines (**B**) the hydrophobic contacts.

The examined biscoumarin derivatives were also docked against AChE using the crystal structure of the *Torpedo californica* AChE in complex with donepezil (pdb 1EVE). To validate the docking protocol, donepezil was re-docked using AutoDock, and the resulting RMSD value between the ligand and the re-docked pose was 1.95 Å, which demonstrated the reliability of the docking protocol. Information about all the docking simulations is reported in the Supplementary Materials (Table S2), whereas Table 3 summarizes the results (i.e., free binding energy and the contacting residues) of some selected ligands, including the most potent AChE inhibitor **3c** identified in the series (Figure 7).

Also in this case, to aid in the interpretation of the docking results, the residues mainly involved in the ligand binding are highlighted in bold, namely Trp84, Phe330, and Phe331 belonging to the anionic site, Phe288 and Phe290 lining the acyl pocket, Gly218, Gly219, and Ala 201 forming the oxyanion hole, and the aromatic residues Trp279, Tyr121, Tyr70, and Tyr334, which together with Asp72 delimit the peripheral anionic site (PAS).

The highest-scored docking pose of donepezil (Figure 7) highlighted the importance of interactions between the dimethoxy phenyl group and Trp279, while the phenyl group demonstrated aromatic interactions with Trp84. The piperidine N attained arene–cation interactions with Trp84 and Phe330. Hydrophobic interactions were found between donepezil and Ile444 and Tyr84.

Compound **3c**, the most potent AChE inhibitor in the series, showed the best docking score (FEB = −9.15 kcal mol^−1^) and the best binding mode (Figure 7). In the best-scored docking pose, **3c** may form an H-bond between C=O of the first coumarin and Tyr70 (2.54 Å distance) and 7-OH and Phe228 (2.87 Å distance). Moreover, hydrophobic contacts and aromatic stackings with Phe330, Phe331, Tyr334, Leu282, Tyr279, and Ile287 may contribute to the stability of the **3c**-AChE complex.

### 2.3. Physicochemical Properties and Drug-likeness Assessment

#### 2.3.1. Water Solubility and Lipophilicity Determinations

Aqueous solubility and lipophilicity were experimentally determined for the five compounds for which finite IC_50_ values could be measured within the threshold concentration close to 10 µM (i.e., **2a**–**d** and **3c**). These compounds include the most potent MAO-A inhibitor (**2b**) and the most potent AChE inhibitor (**3c**); the biscoumarin derivative **3c** achieved, on the other hand, the strongest dual enzymes’ inhibition with single-digit micromolar IC_50_ values. The kinetic solubility in aqueous buffers was determined in HCl (0.01 M, pH 2.0, KCl 0.15 M) and in PBS (50 mM, pH 7.4, KCl 0.15 M) and at 25 ± 1 °C, using RP-HPLC as the analytical method.

In acidic solution (0.01 M HCl), all the tested compounds achieved a kinetic solubility > 2 × 10^−4^ mol·L^−1^, which means that they can be considered from ‘soluble’ to ‘very soluble’ according to the classification of SwissADME based on the topological approach of the ESOL model [34]. In PBS at pH 7.4, the solubilities were found to be two (**2a**) to thirteen (**2c**) to fifty (**3c**) times higher than those calculated with the ESOL model (Table 4), which means that they all fall within the class of ‘soluble’ compounds (−4 < Log *S* < −2) and not within the class of ‘moderately soluble’ compounds (−6 < Log *S* < −4) as predicted by the ESOL-based calculations for neutral molecules.

Most likely, such a strong solubility difference between the calculated and experimental values is due to the different degree of ionization at pH 7.4 with respect to minimum solubility at the isoelectric pH, where these amphoteric molecules should exist as zwitterions. Let us compare the ionization-dependent solubility properties of the homologs **2c** and **3c** as examples.

For the amphoteric compound **2c**, ACD/Labs software (release 9.00. version 9.04) estimates p*K*_a1_ (related to the phenolic OH) and p*K*_a2_ (related to CH_2_NMe_2_) to be about 9.0 and 7.2, respectively, from which an isoelectric pH of 8.1 is calculated. For the NEt_2_ homolog **3c**, the estimated p*K*_a_ values are about 9.4 and 8.2, and hence an isoelectric pH of 8.8. This could imply that at pH 7.4, the calculated protonated fraction of compound **3c** (0.82), and then its aqueous solubility, is consistently higher than that calculated for **2c** (0.14), despite the decrease in hydrophilicity of their neutral forms. Indeed, in contrast with the expected threefold decrease in water solubility, at pH 7.4, the less hydrophilic **3c** achieves a water solubility 1.3-fold higher than that of **2c**.

The lipophilicity of the five compounds in Table 4 was assessed by calculation and experimental approaches. As a calculated lipophilicity parameter (log *P*_calc_), we took from the SwissADME free website (http://www.swissadme.ch) the ‘Consensus Log *P*_o/w_’, that is, the arithmetic mean of octanol/water log *P*s calculated by five methods (iLOGP, XLOGP3, WLOGP, MLOGP, and SILICOS-IT) [34]. To ascertain the consistency of the calculated log *P* scale, a precise and cost-effective RP-HPLC method [36] was used to determine the polycratic capacity factor (log *k*’_w_), that is, the log of the capacity factor (*k*’) extrapolated to 100% water mobile phase, for each compound. The correlation between the RP-HPLC-derived lipophilicity parameter and the predicted log *P*_o/w_ was proven by the following statistically good linear regression Equation (1):

log *k*’_w_ = 0.53 (±0.10) log *P*_calc_ + 0.67 (±0.35) *n* = 5, *R*^2^ = 0.900, *S* = 0.110, *F* = 27.08 (1)

where *R*^2^ is the determination coefficient (i.e., a measure of the explained variance), *S* the standard deviation of the regression model (i.e., a measure of the distance between the observed data points and the regression line), *F* is the F-statistic indicating the overall significance of the regression model, and in parentheses, the 95% confidence intervals of the regression coefficients.

In the above linear equation, the lower-than-unity slope value (+0.53) and the higher-than-zero intercept (+0.67) suggest that retention on the C18 column and partitioning between the immiscible 1-octanol and water are differently influenced by the physicochemical properties of the solutes/analytes. Apparently, the retention of the amphoteric biscoumarins in the RP-HPLC system is affected, not only by the hydrophobic interactions between the long alkyl chains (C18) and the biscoumarin analytes, which in turn dominate in the biphasic partitioning, but also by adsorption and silanophilic interactions, mainly governed by van der Waals interactions, H-bonding, and electrostatic interactions [37]. However, the good statistics associated with the regression equation support the consistency of the relative log *P*_o/w_ scale assessed by the SwissADME website.

Finally, compounds **2c** and **5c**, taken as representative of the series, were evaluated for their hydrolytic stability in 0.01 M HCl_aq_ (KCl 0.15 M, pH 2.0) and in 0.05 M PBS (KCl 0.15 M, pH 7.4) at 37 ± 1 °C. Sample solutions at 100 μM (DMSO as the cosolvent, 1% *v*/*v*) were incubated at 37 ± 1 °C for 1 h, following shaking of the solution on an orbital shaker at 250 rpm and monitoring by RP-HPLC. Both compounds were found to be stable over 1 h at 37 °C in both conditions.

#### 2.3.2. Chemoinformatic Assessment of Drug-likeness and Bioavailability

For the investigated amphoteric biscoumarins achieving finite IC_50_ values less than (or very close to) 10 µM against MAO-A and/or AChE (**2a**–**d**, **3c**, **4d**, **5b**–**d**), the physicochemical properties, including lipophilicity and water solubility, pharmacokinetics (GI absorption, BBB permeation, and potential as P-gp substrate), drug-likeness (as the compliance with the ‘Lipinski’s rule of five’), bioavailability, and alerts for pan-assay interference compounds (PAINS) were estimated by querying the free website SwissADME [34] and are summarized in Table S3 (Supplementary Materials).

According to predictions, all nine active compounds achieved a bioavailability score of 0.55, which should suggest a high probability of efficient absorption, with no violation of Lipinski’s rule of five and moderate aqueous solubility (at pH 7.4, the experimental water solubility resulted in being even higher, as in the case of the most dual active **3c**). The gastrointestinal absorption is estimated to be high, whereas all the compounds are predicted to be ‘not BBB permeant’ and, with two exceptions (the 8′-methoxy congeners **4d** and **5d**), to not be P-gp substrates. While solutions for enhancing their BBB crossing can be found at a more advanced stage of lead optimization, exploiting, for instance, novel prodrug approaches or efficient nanoparticle-based drug delivery systems [38], one issue deals with the PAINS alert related to the presence of the Mannich base at C8 of the first coumarin moiety. This alert requires, for sure, attention in the next stages of study. Nevertheless, it has to be highlighted that several safe drugs in various therapeutic areas, including anti-inflammation, anti-infective, and anticancer areas, bear Mannich base groups [39]. An interesting example of a multitarget-directed ligand (MTDL) as a lead candidate for AD is provided by the *ortho*-phenol Mannich base of the anti-inflammatory flurbiprofen [40], which showed good in vitro BBB permeability, low neurotoxicity, biometal-chelating and antioxidant properties, anti-inflammation, and inhibition of Aβ aggregation and AChE. Other phenolic Mannich bases grafted on privileged scaffolds, such as chalcone [41] or 3-benzylidene/benzylphtalide [42], just to name a few, proved to enhance the multifunctional properties of new MTDLs with potential for treating AD.

## 3. Materials and Methods

### 3.1. Chemistry

#### 3.1.1. General Methods

Starting materials and all chemicals and solvents were purchased from Merck (Darmstadt, Germany). Melting points were determined by using the capillary method on a Stuart Scientific SMP3 electrothermal apparatus (ALT, San Diego, CA, USA) and are uncorrected. Mass spectra were obtained by an Agilent 1100 Series LC–MSD Trap System VL (Agilent Technologies, Santa Clara, CA, USA), equipped with an ESI (electrospray ionization) source, and the high-resolution molecular masses of test compounds were assessed by an Agilent 6530 Accurate Mass Q-TOF spectrometer (Agilent Technologies, Santa Clara, CA, USA). IR spectra (KBr disks) were recorded on a Spectrum One FT infrared spectrophotometer (PerkinElmer, Cambridge, MA, USA), and the most significant absorption bands are listed. ^1^H and ^13^C NMR spectra were recorded at 400 and 101 MHz, respectively, on a Varian Mercury instrument (Agilent Technologies, Santa Clara, CA, USA) in dimethylsulfoxide-*d*_6_ (DMSO-*d*_6_) solution at 25 °C. Chemical shifts data are expressed in *δ* and the coupling constants *J* are in hertz (Hz); the following abbreviations are used for multiplicity: s, singlet; br. s, broad singlet; d, doublet; dd, doublet–doublet; td, triplet of doublets; t, triplet; q, quartet; m, multiplet.

Synthesis and characterization of compounds **1a**–**d** [30,43] have been previously reported.

#### 3.1.2. Synthesis of 8-((Dialkylamino)methyl)-4-(2-oxo-2*H*-chromen-3-yl)-7-hydroxy-2*H*-chromen-2-ones (**2**–**8**)

To a hot solution of a biscoumarin **1a**–**d** (2.0 mmol) in dioxane (10 mL), the suitable aminal (2.2 mmol) was added and the mixture was refluxed for 3–5 h. After TLC control, the reaction mixture was cooled and the solvent evaporated under reduced pressure. Finally, the residue crystallized from the *i*-PrOH–*n*-hexane (1:2, *v*/*v*) mixture.

Synthesis and characterization of compounds **4a**, **5a**–**c**, **6a**, **6b**, **7a**, **7d**, **8a**, and **8b** were published previously [30]. For each newly synthesized compound (**2a**–**d**, **3c**, **4d**, **5d**, **6d**, and **8d**) uncorrected m.p., main IR bands, ^1^H- and ^13^C-NMR data, HRMS *m*/*z* are reported below. ^1^H- and ^13^C-NMR spectra of the newly synthesized compounds (Figures S1–S9), and the HPLC chromatograms of all the tested compounds (Figure S10) are reported in the Supplementary Material.

*8-((dimethylamino)methyl)-4-(2-oxo-2H-chromen-3-yl)-7-hydroxy-2H-chromen-2-one* (**2a**). m.p.: 208–211 °C; ^1^H NMR (400 MHz, DMSO) δ 8.28 (s, 1H), 7.81 (dd, *J* = 7.7, 1.3 Hz, 1H), 7.76–7.68 (m, 1H), 7.51 (d, *J* = 8.2 Hz, 1H), 7.48–7.39 (m, 1H), 7.36 (d, *J* = 8.8 Hz, 1H), 6.67 (d, *J* = 8.8 Hz, 1H), 6.32 (s, 1H), 4.21 (br. s, 1H, OH), 3.96 (s, 2H, CH_2_), 2.38 (s, 6H, (CH_3_)_2_) ppm; ^13^C NMR (101 MHz, DMSO) δ 163.96, 160.39, 159.17, 154.23, 153.15, 151.05, 144.21, 133.21, 129.52, 127.51, 125.28, 123.84, 119.17, 116.74, 113.79, 111.44, 110.03, 108.76, 54.10, 44.37 (2C) ppm; IR (KBr): ν = 3433, 1710, 1737, 1604 cm^−1^; HRMS (Q-TOF): calcd for C_21_H_17_NO_5_ [M − H]^−^ *m*/*z* 362.1034, found 362.1018, [M + Na]^+^ *m*/*z* 386.0999, found 386.1002.

*8-((dimethylamino)methyl)-4-(6-chloro-2-oxo-2H-chromen-3-yl)-7-hydroxy-2H-chromen-2-one* (**2b**). m.p.: 210–212 °C; ^1^H NMR (400 MHz, DMSO) δ 8.21 (s, 1H), 7.92 (d, *J* = 2.6 Hz, 1H), 7.76 (dd, *J* = 8.9, 2.6 Hz, 1H), 7.56 (d, *J* = 8.9 Hz, 1H), 7.39 (d, *J* = 8.8 Hz, 1H), 6.67 (d, *J* = 8.8 Hz, 1H), 3.97 (s, 2H, CH_2_, br. s, 1H, OH), 2.38 (s, 6H, (CH_3_)_2_) ppm;^13^C NMR (101 MHz, DMSO) δ 164.09, 160.29, 158.80, 153.19, 152.88, 150.67, 142.89, 132.68, 128.91, 128.44, 127.57, 125.02, 120.59, 118.77, 113.81, 111.43, 109.88, 108.69, 54.06, 44.33 (2C) ppm; IR (KBr): ν = 3420, 1714, 1604, 1574 cm^−1^; HRMS (Q-TOF): calcd for C_21_H_16_ClNO_5_ [M − H]^−^ *m*/*z* 396.0644, found 396.0642, [M + H]^+^ *m*/*z* 398.0790, found 398.0788, [M + Na]^+^ *m*/*z* 420.0609, found 420.0609.

*8-((dimethylamino)methyl)-4-(6-bromo-2-oxo-2H-chromen-3-yl)-7-hydroxy-2H-chromen-2-one* (**2c**). m.p.: 202–204 °C; ^1^H NMR (400 MHz, DMSO) δ 8.20 (s, 1H), 8.05 (d, *J* = 2.4 Hz, 1H), 7.87 (dd, *J* = 8.8, 2.4 Hz, 1H), 7.50 (d, *J* = 8.8 Hz, 1H), 7.39 (d, *J* = 8.8 Hz, 1H), 6.66 (d, *J* = 8.8 Hz, 1H), 6.31 (s, 1H), 3.96 (s, 2H, CH_2_, br. s, 1H, OH), 2.38 (s, 6H, (CH_3_)_2_) ppm; ^13^C NMR (101 MHz, DMSO) δ 164.12, 160.29, 158.75, 153.30, 153.18, 150.66, 142.81, 135.45, 131.44, 127.55, 124.96, 121.08, 119.03, 116.61, 113.82, 111.42, 109.87, 108.72, 54.10, 44.34 (2C) ppm; IR (KBr): ν = 3435, 1728, 1714, 1601, 1574 cm^−1^; HRMS (Q-TOF): calcd for C_21_H_16_BrNO_5_ [M − H]^−^ *m*/*z* 440.0139, found 440.0124, [M + Na]^+^ *m*/*z* 464.0104, found 464.0194.

*8-((dimethylamino)methyl)-4-(8-methoxy-2-oxo-2H-chromen-3-yl)-7-hydroxy-2H-chromen-2-one* (**2d**). m.p.: 221–225 °C; ^1^H NMR (400 MHz, DMSO) δ 8.26 (s, 1H), 7.52–7.24 (m, 4H), 6.67 (d, *J* = 8.8 Hz, 1H), 6.32 (s, 1H), 3.97 (s, 2H, CH_2_), 3.96 (s, 3H, OCH_3_), 3.78 (br. s, 1H, OH), 2.39 (s, 6H, N (CH_3_)_2_) ppm; ^13^C NMR (101 MHz, DMSO) δ 163.84, 160.35, 158.90, 153.17, 151.02, 146.96, 144.43, 143.59, 127.59, 125.24, 123.99, 120.63, 119.71, 115.45, 113.76, 111.49, 110.05, 108.63, 56.74, 53.96, 44.33 (2C) ppm; IR (KBr): ν = 3430, 1708, 1607, 1384 cm^−1^; HRMS (Q-TOF): calcd for C_22_H_19_NO_6_ [*M* − H]^−^ *m*/*z* 392.1140, found 392.1133, [M + Na]^+^ *m*/*z* 416.1105, found 416.1111.

*8-((diethylamino)methyl)-4-(6-bromo-2-oxo-2H-chromen-3-yl)-7-hydroxy-2H-chromen-2-one* (**3c**). m.p.: 198–200 °C; ^1^H NMR (400 MHz, DMSO) δ 8.20 (s, 1H), 8.05 (d, *J* = 2.4 Hz, 1H), 7.87 (dd, *J* = 8.8, 2.4 Hz, 1H), 7.49 (d, *J* = 8.8 Hz, 1H), 7.36 (d, *J* = 8.8 Hz, 1H), 6.60 (d, *J* = 8.8 Hz, 1H), 6.28 (s, 1H), 4.09 (s, 2H, CH_2_), 3.52 (br. s, 1H, OH), 2.71 (q, *J* = 7.2 Hz, 4H, N-(CH_2_CH_3_)_2_), 1.11 (t, *J* = 7.2 Hz, 6H, N-(CH_2_CH_3_)_2_) ppm; ^13^C NMR (101 MHz, DMSO) δ 160.34, 158.75, 153.29, 153.08, 150.69, 142.76, 135.43, 131.43 (2C), 127.32, 125.01, 121.08, 119.03 (2C), 116.69, 114.10, 109.62, 108.50, 49.43, 46.69 (2C), 11.27 (2C) ppm; IR (KBr): ν = 3433, 1731, 1602, 1378 cm^−1^; HRMS (Q-TOF): calcd for C_23_H_20_BrNO_5_ [M − H]^−^ *m*/*z* 468.0452, found 468.0432, [M + Na]^+^ *m*/*z* 492.0417, found 416.0420.

*8-((dipropylamino)methyl)-4-(8-methoxy-2-oxo-2H-chromen-3-yl)-7-hydroxy-2H-chromen-2-one* (**4d**). m.p.: 128–130 °C; ^1^H NMR (400 MHz, DMSO) δ 8.25 (s, 1H), 7.47–7.27 (m, 4H), 6.62 (d, *J* = 8.8 Hz, 1H), 6.31 (s, 1H), 4.07 (s, 2H, CH_2_), 3.96 (s, 3H, OCH_3_), 3.57 (br., s, 1H, OH), 2.57 (dd, *J* = 8.5, 6.6 Hz, 4H, N-(CH_2_CH_2_CH_3_)_2_), 1.65–1.46 (m, 4H, N-(CH_2_CH_2_CH_3_)_2_), 0.87 (t, *J* = 7.4 Hz, 6H, N-(CH_2_CH_2_CH_3_)_2_) ppm; ^13^C NMR (101 MHz, DMSO) δ 164.24, 160.37, 158.90, 153.02, 151.02, 146.96, 144.42, 143.58, 127.30, 125.24, 124.02, 120.63, 119.71, 115.44, 113.86, 111.38, 109.99, 108.89, 56.74, 55.51 (2C), 50.50, 19.34 (2C), 12.05 (2C) ppm; IR (KBr): ν = 3430, 2962, 1712, 1605, 1478,1379 cm^−1^; HRMS (Q-TOF): calcd for C_26_H_27_NO_6_ [M − H]^−^ *m*/*z* 448.1766, found 448.1744, [*M* + Na]^+^ *m*/*z* 472.1731, found 472.1741.

*8-((butyl(methyl)amino)methyl)-4-(8-methoxy-2-oxo-2H-chromen-3-yl)-7-hydroxy-2H-chromen-2-one* (**5d**). m.p.: 163–165 °C; ^1^H NMR (400 MHz, DMSO) δ 8.25 (s, 1H), 7.46–7.27 (m, 4H), 6.63 (d, *J* = 8.8 Hz, 1H), 6.31 (s, 1H), 4.01 (s, 2H, CH_2_), 3.96 (s, 3H, OCH_3_), 3.54 (br. s, 1H, OH), 2.64–2.54 (m, 2H, N-(CH_2_CH_2_CH_2_CH_3_)), 2.32 (s, 3H, N-CH_3_), 1.54 (dt, *J* = 14.9, 7.4 Hz, 2H, N-(CH_2_CH_2_CH_2_CH_3_)), 1.32 (dq, *J* = 14.5, 7.3 Hz, 2H, N-(CH_2_CH_2_CH_2_CH_3_)), 0.90 (t, *J* = 7.3 Hz, 3H, N-(CH_2_CH_2_CH_2_CH_3_)) ppm; ^13^C NMR (101 MHz, DMSO) δ 160.38, 158.90, 151.02, 146.96, 144.40 (2C), 143.59, 127.40, 125.23 (2C), 124.03, 120.63 (2C), 119.71, 115.44, 113.86, 108.68 (2C), 56.74, 56.37, 53.07, 41.24, 28.66, 20.26, 14.22 ppm; IR (KBr): ν = 3437, 2935, 1720, 1603 cm^−1^; HRMS (Q-TOF): calcd for C_25_H_25_NO_6_ [M − H]^−^ *m*/*z* 434.1609, found 434.1618, [M + Na]^+^ *m*/*z* 454.1574, found 454.1586.

*8-((bis(2-methoxyethyl)amino)methyl)-4-(8-methoxy-2-oxo-2H-chromen-3-yl)-7-hydroxy-2H-chromen-2-one* (**6d**). m.p.: 165–167 °C; ^1^H NMR (400 MHz, DMSO) δ 8.26 (s, 1H), 7.54–7.13 (m, 4H), 6.68 (d, *J* = 8.8 Hz, 1H), 6.35 (s, 1H), 4.12 (s, 2H, CH_2_), 3.96 (s, 3H, OCH_3_), 3.51 (t, *J* = 5.5 Hz, 4H, (NCH_2_-CH_2_OCH_3_)_2_), 3.24 (s, 6H, (NCH_2_-CH_2_OCH_3_)_2_), 2.81 (t, *J* = 5.5 Hz, 4H, (NCH_2_-CH_2_OCH_3_)_2_) ppm; ^13^C NMR (101 MHz, DMSO) δ 162.97, 160.33, 158.89, 153.05, 151.00, 146.97, 144.46, 143.60, 127.34, 125.24, 123.97, 120.64, 119.71, 115.46, 113.63, 111.95, 110.50, 109.75, 69.63 (2C), 58.50 (2C), 56.74, 53.18 (2C), 49.82 ppm; IR (KBr): ν = 3437, 1725, 1600, 1384 cm^−1^; HRMS (Q-TOF): calcd for C_26_H_27_NO_8_ [M − H]^−^ *m*/*z* 480.1664, found 480.1649, [M + Na]^+^ *m*/*z* 504.1629, found 504.1638.

*8-(((benzyl(methyl)amino)methyl)-4-(8-methoxy-2-oxo-2H-chromen-3-yl)-7-hydroxy-2H-chromen-2-one* (**8d**). m.p.: 204–206 °C; ^1^H NMR (400 MHz, DMSO) δ 8.26 (s, 1H), 7.49–7.22 (m, 9H), 6.71 (d, *J* = 8.8 Hz, 1H), 6.36 (s, 1H), 4.02 (s, 2H), 3.96 (s, 3H, OCH_3_), 3.74 (s, 2H, Bn), 2.23 (s, 3H, NCH_3_) ppm; ^13^C NMR (101 MHz, DMSO) δ 162.73, 160.31, 158.88, 153.20, 150.97, 146.97, 144.48, 143.60, 137.50, 129.73 (2C), 128.94 (2C), 127.99, 127.54, 125.24, 123.95, 120.64, 119.71, 115.46, 113.48, 112.04, 110.59, 109.38, 60.97, 56.74, 51.84 ppm; IR (KBr): ν = 3439, 1723, 1602 cm^−1^; HRMS (Q-TOF): calcd for C_28_H_23_NO_6_ [*M* − H]^−^ *m/z* 468.1453, found 468.1451, [M + Na]^+^ *m*/*z* 492.1418, found 504.1413.

### 3.2. Determination of Kinetic Aqueous Solubility and Lipophilicity

Water solubility and lipophilicity parameters were measured following previously reported methods [36]. For measuring kinetic solubility in aqueous buffers, a 200 µM sample solution in PBS (50 mM, pH 7.4, KCl 0.15 M), or in HCl (0.01 M, pH 2.00, KCl 0.15 M) prepared from a 10 mM stock solution in DMSO, was incubated at room temperature (25 ± 1 °C) for 2 h while being continuously shaken at 250 rpm on an orbital shaker. The mixture was then separated by centrifugation at 2500 rpm for 3 min. Immediately after filtration, 100 µL of the filtrate was mixed with an equal volume of a 1:1 (*v*/*v*) DMSO and PBS (50 mM, pH 7.4, KCl 0.15 M) or HCl (0.01 M, pH 2, KCl 0.15 M) solution to prevent precipitation from the saturated solution and subsequently analyzed by HPLC. The peak area was compared to a calibration curve of the tested compound in MeOH. The analytical parameters were set as follows. Mobile phase: MeOH/water pH 2.8 (acetic acid 0.5% *v*/*v*) at different percentages depending on the retention time of each test compound; stationary phase: Phenomenex, Kinetex 5 µ, C8, (150 × 3 mm); flux: 0.5 µL/min; injection: 2 µL; λ 330 nm. The analyses were performed on the Agilent HPLC 1260 Infinity Series Integrated System (Agilent Technologies, Milan, Italy).

The lipophilicity parameters were determined by an RP-HPLC technique [36]. In short, methanolic solutions of the examined compounds (1 mg·mL^−1^) were analyzed by the Agilent 1260 infinite HPLC system (Agilent Technologies, Milan, Italy) equipped with a diode array detector (DAD), using a Phenomenex C18-column (Kinetex 5 μ, 100 Å, 150 × 3 mm) as the stationary phase and MeOH/water buffer mobile phases with different *v*/*v* composition (i.e., mobile phases containing 0.05 increments of MeOH volume fractions in 10 mM ammonium formate buffer at pH 4.5; φ_MeOH_ ranging between 0.70 and 0.20). The chromatographic measurements were carried out at 25 ± 1 °C, flow rate of 0.5 mL·min^−1,^ and detection at λ 330 nm. The log of capacity factors of each compound at different mobile phase compositions was calculated (log *k*′ = log (*t*_R_ − *t*_0_)/*t*_0_, where *t*_R_ represents the retention time of the solute and *t*_0_ is the column dead time). For each compound, the equation of the linear relationship between log *k*′ and φ_MeOH_ (*R*^2^ > 0.991) on at least five datapoints, each from duplicate, was derived by regression analysis and the intercept at the *y*-axis (i.e., φ_MeOH_ = 0) calculated as the lipophilicity parameter log *k*′_w_.

### 3.3. Enzyme Inhibition Assays

#### 3.3.1. Inhibition of Monoamine Oxidases

The inhibition assays of human monoamine oxidases were conducted using a fluorescence-based method with kynuramine as a non-selective substrate for each isoform, MAO-A/B [44,45,46]. Recombinant human MAO-A and MAO-B (microsomes from insect cells infected with baculovirus; Sigma Aldrich, St. Louis, MO, USA) (Merk Life Science S.r.l., Milan, Italy) were used. The measurements were performed in triplicate in black round-bottom polystyrene 96-well plates (Greiner Bio-One, Kremsmünster, Austria). Fluorescence readings were performed using an Infinite M1000 Pro plate reader (Tecan, Cernusco sul Naviglio, Milano, Italy), and inhibition values were calculated using Prism (version 5.01 for Windows; GraphPad Software, San Diego, CA, USA). To evaluate enzymatic activity, clorgiline (a selective MAO-A inhibitor) and selegiline (a selective MAO-B inhibitor) were employed as positive controls. In the MAO inhibition assays, all compounds were initially screened at a concentration of 10 µM. Each of them was preincubated at 37 °C for 20 min with 50 µM kynuramine in a phosphate buffer (PBS, 0.1 M, pH 8.0), adjusted to 0.39 osmolarity with KCl. After adding recombinant human MAO-A/B (250 U/mg and 59 U/mg, respectively) and incubating for another 30 min, NaOH was added. The fluorescence was measured at excitation/emission wavelengths of 310/400 nm. Compounds showing at least 60% MAO inhibition at 10 µM were further tested at seven different concentrations to determine the IC_50_ value, calculated via nonlinear regression. The IC_50_ is reported as the mean ± SD of three independent experiments, each performed in duplicate.

#### 3.3.2. Inhibition of Cholinesterases

Cholinesterase inhibition assays were carried out using the Ellman spectrophotometric method, modified for a 96-well plate format [45,47]. Acetylcholinesterase from electric eel and butyrylcholinesterase from equine serum (Sigma Aldrich) were employed. The measurements were performed in triplicate using clear, flat-bottom polystyrene plates (Greiner Bio-One). To assess enzymatic activity, donepezil (AChE-selective) and tacrine (BChE-selective) were used as positive controls. The solutions containing AChE (1.2 U/mL) or BChE (2 U/mL), 5,5′-dithiobis(2-nitrobenzoic acid) (Ellman’s reagent, 0.33 mM), the test compound (10 µM concentration for the initial screening, or seven different concentrations for compounds achieving > 60% enzyme inhibition at 10 µM to determine the IC_50_ value), in PBS (0.1 M, pH 8.0), were incubated for 20 min at 25 °C. Then, acetylthiocholine or butyrylthiocholine iodide (5 µM) as a substrate was added, and the hydrolysis rates were recorded for 5 min at 412 nm. The IC_50_ value, calculated using the nonlinear regression method ‘log[inhibitor] vs. response’, or % inhibition at 10 µM, is reported as the mean ± SD of three independent experiments, each performed in duplicate.

### 3.4. Molecular Docking Calculations

To comprehend the molecular binding mechanisms of the active compounds toward two distinct biological targets that are associated with human monoamine oxidase enzymes, molecular docking studies were conducted. The human MAO-A (PDB code 2BXR) and human MAO-B (PDB code 1GOS) crystallographic structures from the Protein Data Bank [48] (PDB) were taken into consideration as the enzyme targets for the docking simulations. In the docking simulations, the proteins were considered without co-crystallized ligands. The docking protocol was validated by performing the simulation with only the bound ligands and low RMSD between the docked and crystal conformations, prior to screening the active compounds.

Specifically, the FAD cofactor was retained in the binding sites of both MAO-A and MAO-B to preserve the integrity of the catalytic pocket. In addition, the docking protocol was validated by re-docking the co-crystallized ligands (clorgyline for MAO-A and safinamide for MAO-B), obtaining RMSD values < 2.0 Å between the docked and crystallographic geometries, which confirmed the reliability of the docking setup. The quality of the structural models of the target enzymes was assessed using Ramachandran plots generated by MolProbity (http://molprobity.biochem.duke.edu/help/about.html, accessed on 13 February 2025). For MAO-A (PDB 2BXR), 89.2% of residues (783/878) were in favored regions, while 98.1% (861/878) fell within allowed regions. A total of 17 residues were identified as outliers, mainly clustered in loop segments (e.g., residues 61, 107–109, 118, 215, and 252 in both chains) and including a single glycine (428) and glutamate (399). Outliers in proline and glycine are not uncommon due to their conformational flexibility, and in this structure, they are positioned away from the active site, with no effect on the overall fold or the geometry of the catalytic pocket. The binding environment around the FAD cofactor and the substrate cavity remains well defined, supporting its suitability for molecular docking. In contrast, acetylcholinesterase (PDB 1EVE) exhibited higher stereochemical quality, with 94.2% of residues (501/532) in favored regions and 100% (532/532) in allowed regions, and no outliers detected. Taken together, these results indicate that while MAO-A shows a modest fraction of outliers compared to AChE, the deviations are restricted to flexible, non-critical regions, and the structural quality is sufficient to provide a reliable receptor model for docking studies.

The ligands’ structures were prepared by drawing them in Chemdraw 12.0 software. All docking calculations were performed using AutoDock 4.2 [46] software. The docking input files were created, and the docking outcomes were analyzed using the AutoDock Tools software. A 90 × 90 × 90 grid box was created, covering nearly the whole surface of the protein, with grid points spaced 0.375 Å apart. Before the computations, all non-polar hydrogens and crystallographic water molecules were eliminated. The mass center of the TSA that was bound served as the docking grid’s center. Using genetic algorithm searches, 100 docked structures were produced in each case.

A default protocol was used, with an initial population of 50 randomly arranged conformations. Heavy atom comparison root mean square deviations (RMSD values) were determined and initial ligand binding modes were plotted. Plots of protein–ligand interactions were generated using LigandScout software (vers. 2.0) [49].

### 3.5. Drug-likeness and Bioavailability Assessment

Physicochemical properties, such as the topological polar surface area (TPSA), consensus logarithm of partition coefficient (Log *P*_o/w_), and water solubility (Log *S*), pharmacokinetics, ADME-related features, and drug-likeness of the investigated compounds were estimated using the free web server SwissADME [34].

## 4. Conclusions

Evaluating the inhibitory activity of MAOs and ChEs of nineteen amphoteric 3,4′-biscoumarins bearing a (dialkylamino)methyl group at C8 and a phenolic hydroxyl at C7 of the coumarin core, some compounds proved to act as moderate inhibitors of MAO-A and/or AChE with a single-digit micromolar IC_50_. Structure–activity relationships (SARs) suggested that the enzymes’ inhibitory potency depends upon the bulkiness and hydrophobicity of the tertiary amino head of the *ortho*-phenolic Mannich base. Indeed, the highest inhibition potency against MAO-A (IC_50_ = 1.49 µM) was achieved by the 8-(dimethylamino)methyl derivative **2b** (6′-Cl congener), whereas the 8-(diethylamino)methyl derivative **3c** (6′-Br congener) exhibited the highest inhibition potency against AChE (IC_50_ = 1.56 µM) as well as a good inhibition of MAO-A (IC_50_ = 3.04 µM).

Even though MAO-B inhibition is recognized as a mechanism contributing to treating PD and AD, selective MAO-A inhibitors could be used to treat depressive forms often associated with the early phases of cognitive decline related to AD [50]. In this study, molecular docking calculations provided support in understanding the key interactions responsible for MAO-A inhibition. The biscoumarin derivative **2b** binds into the MAO-A active pocket in a mode like that adopted by the reference MAO-A selective reversible inhibitor moclobemide forming H-bond interactions with Ser209 and Tyr444, as well as hydrophobic/aromatic interactions; the binding affinity of **2b** is favored by an almost parallel π-stacking between the coumarin-3-yl moiety and the flavin of the cofactor FAD. The most potent AChE inhibitor **3c** in this series achieved the best docking score, but a binding mode quite different from that of the reference drug donepezil. The most potent dual inhibitor **3c**, which can be taken as a hit for future molecular optimization studies and candidate for further AD-related targeted biochemical and biophysical assays (e.g., biometal-chelating, antioxidant, β-amyloid aggregation), showed a good water solubility at pH 7.4 (1.8 mol·L^−1^; fiftyfold more soluble than calculated). Chemoinformatics suggests for **3c** full compliance with Lipinski’s rule of five, high probability of gastrointestinal absorption, but low BBB permeation. While further efforts are required to optimize their BBB permeability [51], herein we identified novel phenolic Mannich bases of 4-(2-oxo-2*H*-chromen-3-yl)-2*H*-chromen-2-one as potential MTDLs for treating AD and neurodegenerative syndromes. To this aim, new experiments will be carried out, including Aβ/tau protein aggregation and cell-based neuroprotection assays, on new derivatives identified in the ongoing molecular optimization study, whose results will be reported in due course.

## Figures and Tables

**Table 1:** Monoamine oxidases (MAO A and B) and cholinesterases (AChE and BChE) inhibition data of 8-[(dialkylamino)methyl]-4-(2-oxo-2*H*-chromen-3-yl)-7-hydroxy-2*H*-chromen-2-one derivatives ^a^.

Cmpd	NR_2_	R^1^	R^2^	*hu*MAO-A	*hu*MAO-B	*ee*AChE	*hu*AChE	*eq*BChE
**2a**	NMe_2_	H	H	**8.30 ± 0.36**	[36 ± 5]	[22 ± 3]		i.a.
**2b**		Cl	H	**1.49 ± 0.49**	~10	~10	i.a.	i.a.
**2c**		Br	H	**2.23 ± 0.22**	~10	[20 ± 1]	i.a.	i.a.
**2d**		H	OMe	**11.7 ± 1.3**	[28 ± 3]	[39 ± 9]		[25 ± 3]
**3c**	NEt_2_	Br	H	**3.04 ± 1.37**	[42 ± 3]	**1.56 ± 0.31**	**4.27 ± 0.58**	[37 ± 4]
**4a**	N(nPr)_2_	H	H	[29 ± 6]	[33 ± 1]	[37 ± 3]		[22 ± 3]
**4d**		H	OMe	[29 ± 5]	[43 ± 5]	**3.05 ± 0.97**	**0.97 ± 0.20**	[14 ± 10]
**5a**	N(Me)nBu	H	H	[32 ± 3]	[23 ± 3]	[28 ± 3]		[27 ± 2]
**5b**		Cl	H	**1.85 ± 0.11**	~10	[25 ± 5]	~10	[17 ± 7]
**5c**		Br	H	**2.52 ± 0.70**	~10	**4.25 ± 0.5**		[25 ± 11]
**5d**		H	OMe	[39 ± 4]	~10	**7.80 ± 1.92**		[40 ± 4]
**6a**	N(CH_2_CH_2_OMe)_2_	H	H	[32 ± 5]	[29 ± 6]	[37 ± 9]		[15 ± 4]
**6b**		Cl	H	[45 ± 3]	[35 ± 9]	[16 ± 4]		i.a.
**6d**		H	OMe	[26 ± 4]	[31 ± 1]	[20 ± 2]		i.a.
**7a**	N(*i*Bu)_2_	H	H	[27 ± 2]	[28 ± 4]	~10		~10
**7d**		H	OMe	[27 ± 3]	[31 ± 2]	~10		[34 ± 3]
**8a**	N(Me)Bn	H	H	[28 ± 4]	~10	[40 ± 9]		[31 ± 4]
**8b**		Cl	H	~10	[38 ± 3]	[16 ± 6]		i.a.
**8d**		H	OMe	[31 ± 4]	[42 ± 3]	~10		~10
Clorgiline				0.0025 ± 0.0002	2.51 ± 0.45			
Donepezil						0.051 ± 0.003		2.71 ± 0.31

^a^ Enzymes are human *hu*MAOs A and B, electric eel *ee*AChE, and equine (horse serum) *eq*BChE. IC_50_ values (µM, bold) are listed for compounds achieving more than 60% inhibition at 10 µM concentration or % inhibition at 10 µM concentration in square brackets, expressed as mean ± SD of three independent determinations; IC_50_ reported as ~10 for compounds achieving at 10 µM concentration average % inhibition within the range 50–60%; i.a., compounds displaying average % inhibition less than 10% at 10 µM concentration.

**Table 2:** Molecular docking results with MAO-A (pdb 2BXR).

Cmpd	Free Energy of Binding (kcal·mol^−1^) ^a^	H-Bonding ^b^	Hydrophobic/ Aromatic Interactions ^b^	Ionic Interactions ^b^
**2a**	−11.38	Ile207, Ser209, Tyr447	**Phe208**, Leu337, Tyr407, **Tyr444**	Glu216
**2b**	−11.25	Ser209, **Tyr444**	Ala68, **Tyr407**, The352, **Phe208**, Tyr68, Met350, Ile180, Val93, **Ile335**	-
**2d**	−7.62	Met445	Thr52, **Tyr44**	-
**4a**	−10.06	Tyr69, Ser209, **Tyr407**	**Phe208**, Val303, **Ile335**, Phe352, **Tyr444**, Met445	**Tyr407**
**5a**	−7.82	Ser209, Ile207	Leu97, **Phe208**, Leu337	-
**5b**	−10.98	Ser209, **Tyr444**	Tyr68, Ala68, Ile180, **Phe208**, Met350, **Ile335**	-
Moclobemide	−8.92	Ser209, **Tyr444**	Leu97, **Phe208**, Ile325, **Ile335**, Leu337	-

^a^ Free energy of binding (kcal·mol^−1^) estimated by AutoDock 4.2 software for ligands in the highest-scored binding pose. ^b^ Residues in the binding site of MAO-A mainly involved in the interaction with biscoumarin ligands; residues established as playing a role in substrate and inhibitor selectivity are highlighted in bold.

**Table 3:** Molecular docking results with AChE (pdb 1EVE).

Cmpd	Free Binding Energy (kcal·mol^−1^) ^a^	H-Bonding	Hydrophobic/Aromatic Interactions ^b^	Ionic Interactions ^b^
**2a**	−5.12	-	**Phe331**, **Tyr334**	-
**2c**	−2.13	-	-	-
**3c**	−9.15	Tyr70, Phe228	**Phe330**, **Phe331**, **Tyr334**, Leu282, **Trp279**, Ile287, **Phe290**	-
**4d**	−7.15	Tyr70	**Trp279**, **Phe290**	-
**5c**	−8.02	-	**Trp84**, **Trp279**, **Phe290**	-
**5d**	−8.51	**Trp84**	**Trp84**, **Tyr279**, Ile287, **Phe290**	-
Donepezil	−9.53	-	**Trp84**, Ile144	**Phe330**, **Trp84**

^a^ Free energy of binding (kcal·mol^−1^) estimated by AutoDock 4.2 software for ligands in the highest-scored binding pose. ^b^ Residues in the binding site of AChE mainly involved in the interaction with biscoumarin ligands. Residues lining the anionic site, acyl pocket, oxyanion hole, and peripheral anionic site, which are well-established as playing a role in substrate and inhibitor selectivity, are highlighted in bold.

**Table 4:** Calculated and experimental solubility and lipophilicity data.

Cmpd	Solubility (mol/L)		Lipophilicity	
	Calc. ^a^	Exp. ^b^	Log P_calc_ ^c^	Log k’_w_ ^d^
**2a**	8.79 × 10^−5^	1.83 × 10^−4^	2.96	2.11
**2b**	2.26 × 10^−5^	1.47 × 10^−4^	3.45	2.45
**2c**	1.08 × 10^−5^	1.41 × 10^−4^	3.58	2.65
**2d**	7.49 × 10^−5^	1.96 × 10^−4^	2.94	2.32
**3c**	3.69 × 10^−6^	1.81 × 10^−4^	4.26	2.89

^a^ Solubility calculated by chemoinformatic tools of the SwissADME free website (http://www.swissadme.ch, accessed on 27 June 2025), applying the topological method (ESOL) implemented by J.S. Delaney [35]. ^b^ Average solubility (n = 3, RSD < 5%) determined in PBS (50 mM, pH 7.4, 0.15 M KCl) at 25 ± 1 °C. ^c^ Consensus Log P_o/w_ calculated by SwissADME as the arithmetic mean of octanol/water log Ps calculated by five methods (iLOGP, XLOGP3, WLOGP, MLOGP, and SILICOS-IT), as reported in ref. [34]. ^d^ Log of the polycratic capacity factor (k’_w_) determined as lipophilicity parameter by a reversed-phase (RP) HPLC technique [36].

## Data Availability

The original contributions presented in this study are included in the article and Supplementary Materials. Further inquiries can be directed to the corresponding authors.

## Supplementary Materials

The supporting information can be downloaded at: https://www.mdpi.com/article/10.3390/ijms262010197/s1.
