# Supplementary material for: Investigating Amphoteric 3,4′-Biscoumarin-Based ortho-[(Dialkylamino)methyl]phenols as Dual MAO and ChE Inhibitors
Source: Int J Mol Sci. 2025 Oct 20;26(20):10197. doi: 10.3390/ijms262010197 (PMC12562640; doi:10.3390/ijms262010197)
Supplement: Supplementary file 1 [file ijms-26-10197-s001.zip › ijms-3824856-supplementary.pdf]

Investigating amphoteric 3,4′-biscoumarin-based *ortho*-[(dialkylamino)methyl]phenols as dual MAO and ChE inhibitors

Anthi Petrou [1,¶], Caterina Deruvo [2,¶], Rosa Purgatorio [2], Boris Lichitsky [3], Andrey N. Komogortsev [3], Victor G. Kartsev [4], Modesto de Candia [2], Marco Catto [2], Cosimo D. Altomare [2,*] and Athina Geronikaki [1,*]

[1] Department of Pharmaceutical Chemistry, School of Pharmacy, Aristotle University of Thessaloniki, 54124 Thessaloniki (Greece); anthi.petrou.thessalonik1@gmail.com (A.P.);

[2] Department of Pharmacy-Pharmaceutical Sciences, University of Bari Aldo Moro, Via E. Orabona 4, 70125 Bari (Italy); caterina.deruvo@uniba.it (C.D.); rosa.purgatorio@uniba.it (R.P.); modesto.decandia@uniba.it (M.D.C.); marco.catto@uniba.it (M.C.);

[3] Zelinsky Institute of Organic Chemistry, Leninsky prospect, 119991, Moscow, Russian Federation; blich2006@mail.ru (B.L.); dna5@mail.ru (A.N.K.);

[4] InterBioScreen Ltd., Moscow 119019, Russian Federation; vkartsev@ibscreen.chg.ru (V.G.K.);

* Correspondence: (C.D.A.) cosimodamiano.altomare@uniba.it ; tel.: +39-080-5442781;
(A.G.) geronik@pharm.auth.gr ; tel.: +30-231-0997616

¶ Equally contributing authors

SUPPLEMENTARY MATERIAL

Contents

Figure S1. ^{1}H- and ^{13}C-NMR spectra of 8-((dimethylamino)methyl)- 4-(2-oxo-2*H*-chromen-3-yl)-7-hydroxy-2*H*-chromen-2-one (**2a**) **3**

Figure S2. ^{1}H- and ^{13}C-NMR spectra of 8-((dimethylamino)methyl)-4-(6-chloro-2-oxo-2*H*-chromen-3-yl)-7-hydroxy-2*H*-chromen-2-one (**2b**) **4**

Figure S3. ^{1}H- and ^{13}C-NMR spectra of 8-((dimethylamino)methyl)-4-(6-bromo-2-oxo-2*H*-chromen-3-yl)-7-hydroxy-2*H*-chromen-2-one (**2c**) **5**

Figure S4. ^{1}H- and ^{13}C-NMR spectra of 8-((dimethylamino)methyl)-4-(8-methoxy-2-oxo-2*H*-chromen-3-yl)-7-hydroxy-2*H*-chromen-2-one (**2d**) **6**

Figure S5. ^{1}H- and ^{13}C-NMR spectra of 8-((diethylamino)methyl)-4-(6-bromo-2-oxo-2*H*-chromen-3-yl)-7-hydroxy-2*H*-chromen-2-one (**3c**) **7**

Fugure S6. ^{1}H- and ^{13}C-NMR spectra of 8-((dipropylamino)methyl)-4-(8-methoxy-2-oxo-2*H*-chromen-3-yl)-7-hydroxy-2*H*-chromen-2-one (**4d**) **8**

Figure S7. ^{1}H- and ^{13}C-NMR spectra of 8-((butyl(methyl)amino)methyl)-4-(8-methoxy-2-oxo-2*H*-chromen-3-yl)-7-hydroxy-2*H*-chromen-2-one (**5d**) **9**

Figure S8. ^{1}H- and ^{13}C-NMR spectra of 8-((bis(2-methoxyethyl)amino)methyl)-4-(8-methoxy-2-oxo-2*H*-chromen-3-yl)-7-hydroxy-2*H*-chromen-2-one (**6d**) **10**

Figure S9. ^{1}H- and ^{13}C-NMR spectra of 8-(((benzyl(methyl)amino)methyl)-4-(8-methoxy-2-oxo-2*H*-chromen-3-yl)-7-hydroxy-2*H*-chromen-2-one (**8d**) **11**

Figure S10. RP-HPLC chromatograms of the tested bis-coumarin derivatives **12**

Figure S11. Time-dependent inhibition of human MAO-A by compounds **2b**, **5b** and clorgiline **12**

Table S1. Molecular docking results with monoamine oxidases (MAOs A and B) **18**

Table S2. Molecular docking results with acetylcholinesterase (AChE) **19**

Table S3. SwissADME-assessed of the main physicochemical properties, pharmacokinetics, drug-likeness and bioavailability scores of the tested amphoteric bis-coumarin derivatives **20**

^{1}H- and ^{13}C-NMR spectra of the newly synthesized compounds 2a-2d, 3c, 4d, 5d, 6d, 8d

Figure S1. 8-((dimethylamino)methyl)- 4-(2-oxo-2*H*-chromen-3-yl)-7-hydroxy-2*H*-chromen-2-one (**2a**)

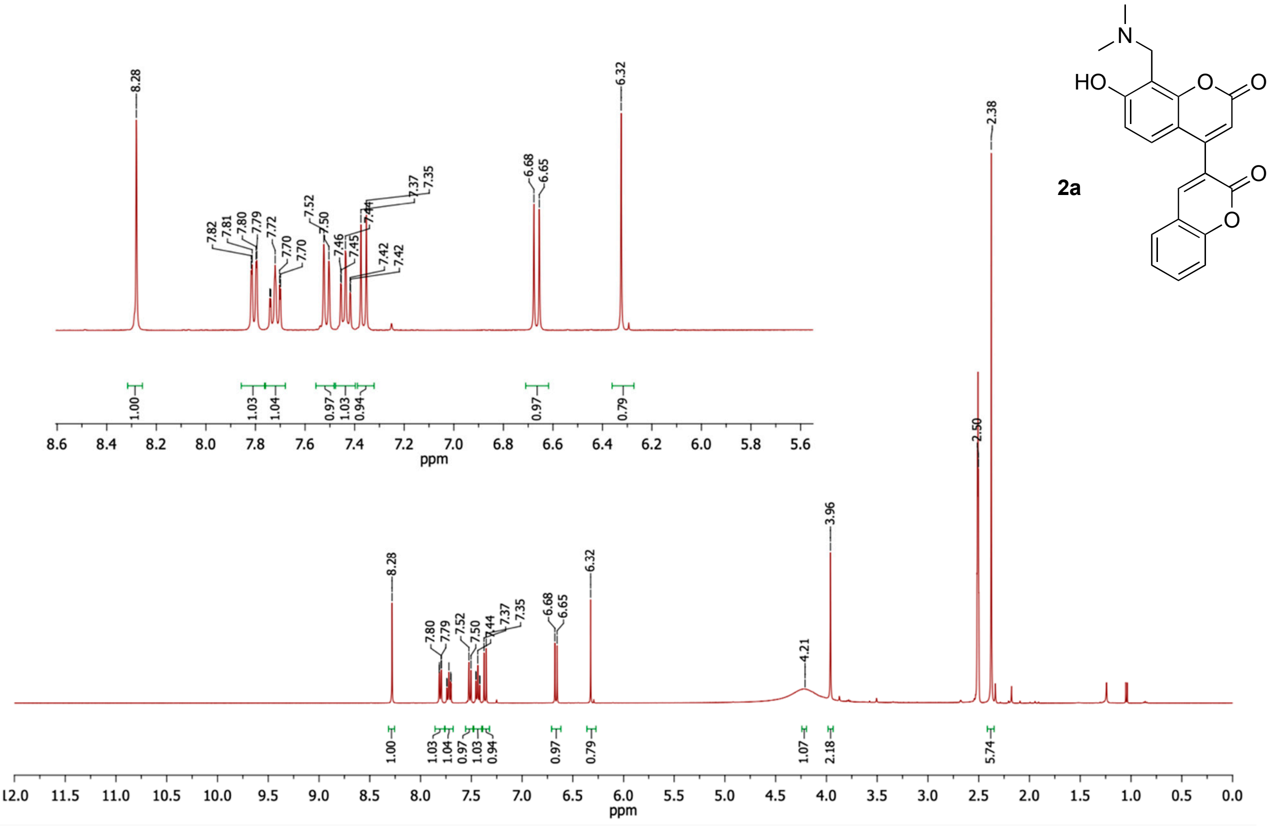

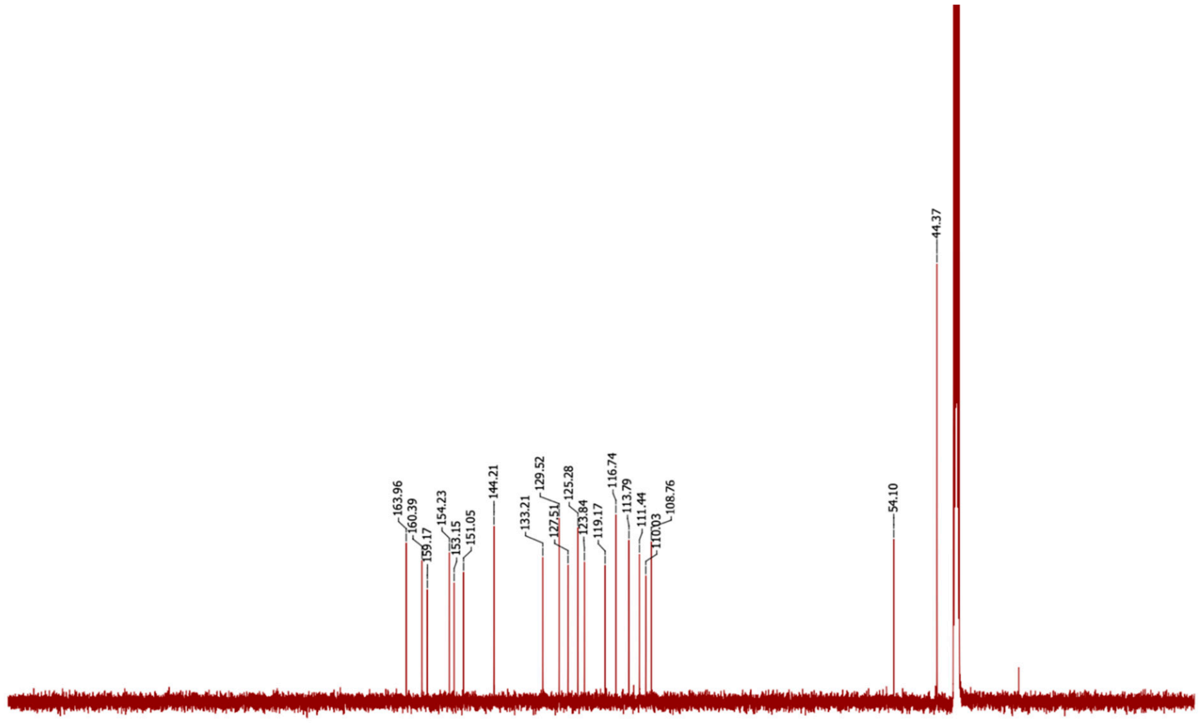

Figure S2. 8-((dimethylamino)methyl)-4-(6-chloro-2-oxo-2H-chromen-3-yl)-7-hydroxy-2H-chromen-2-one (**2b**)

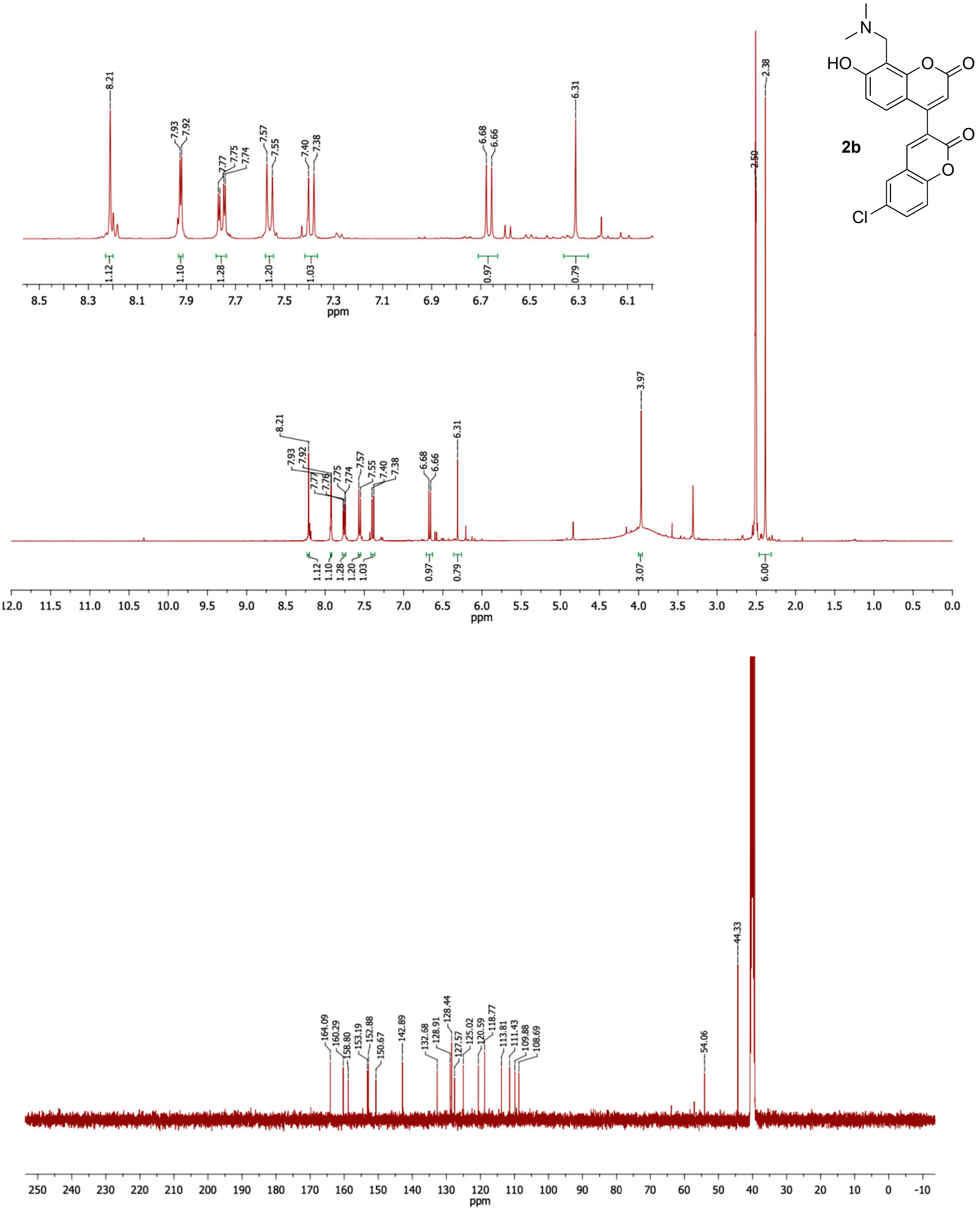

Figure S3. 8-((dimethylamino)methyl)-4-(6-bromo-2-oxo-2*H*-chromen-3-yl)-7-hydroxy-2*H*-chromen-2-one **(2c)**

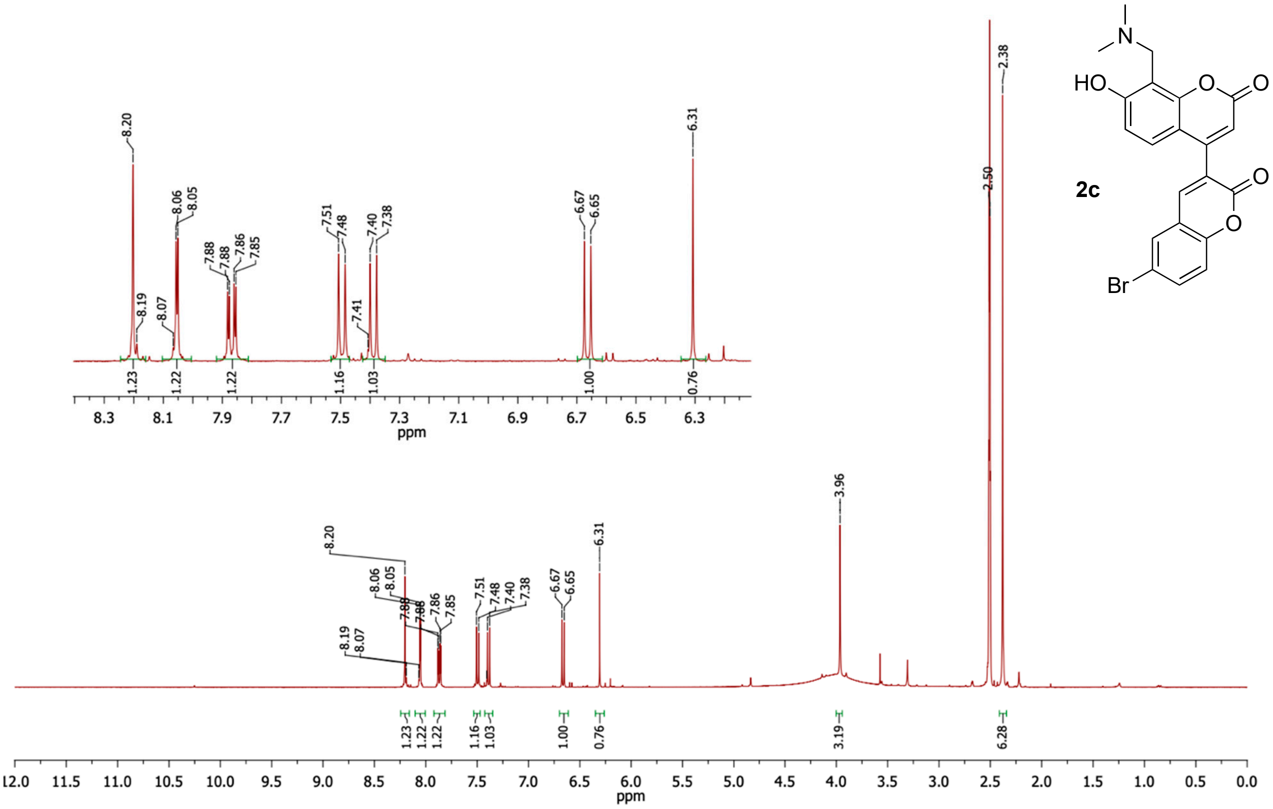

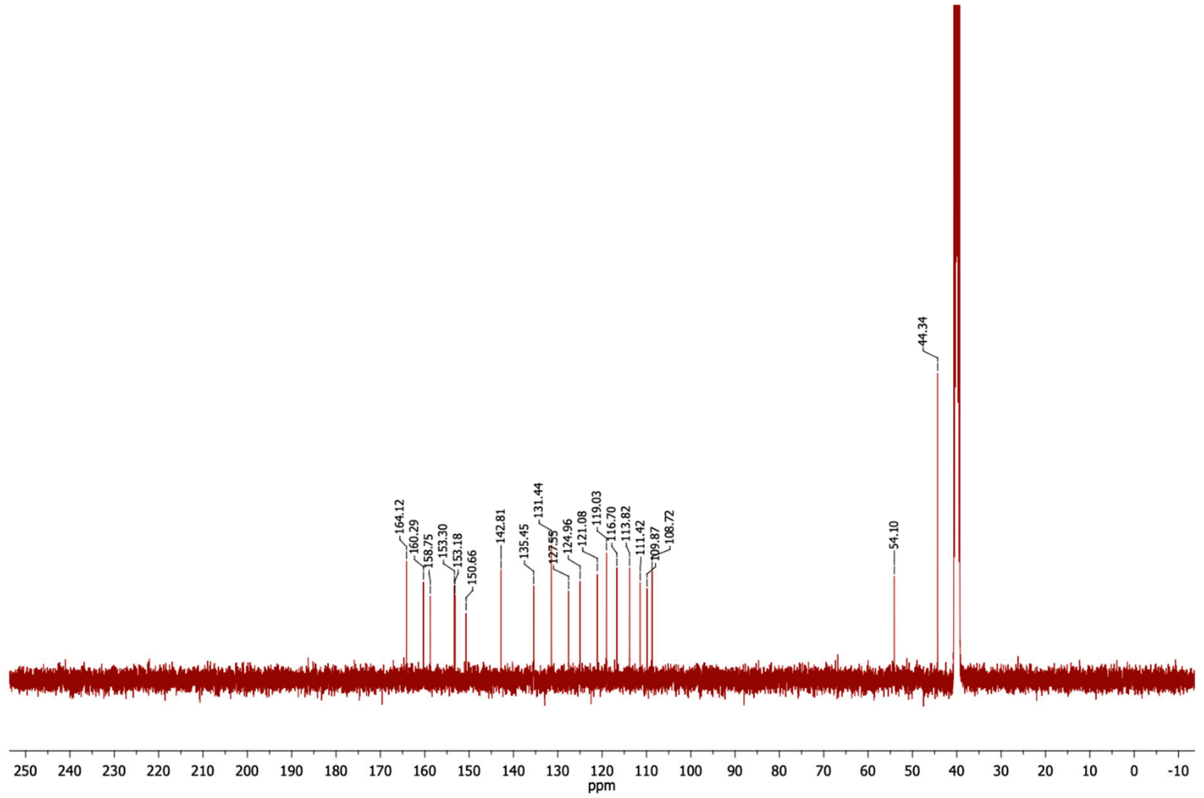

Figure S4. 8-((dimethylamino)methyl)-4-(8-methoxy-2-oxo-2*H*-chromen-3-yl)-7-hydroxy-2*H*-chromen-2-one (**2d**)

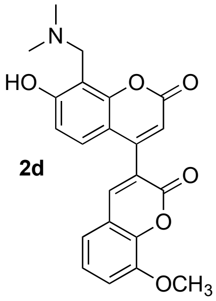

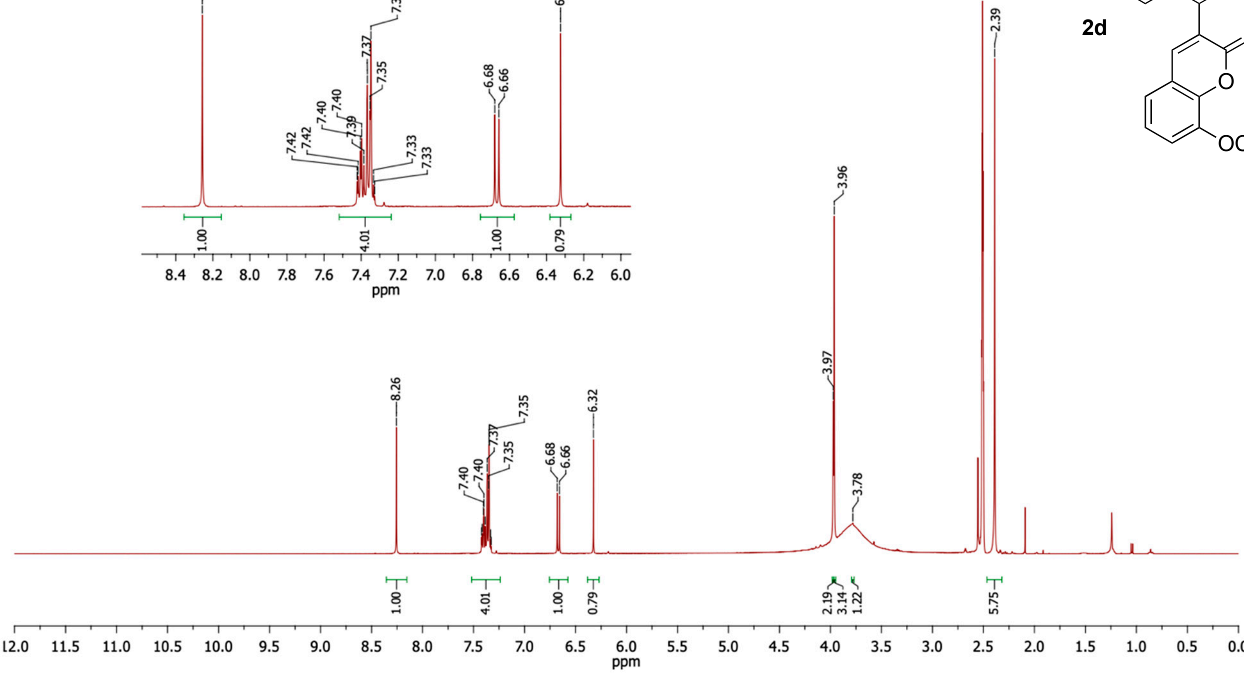

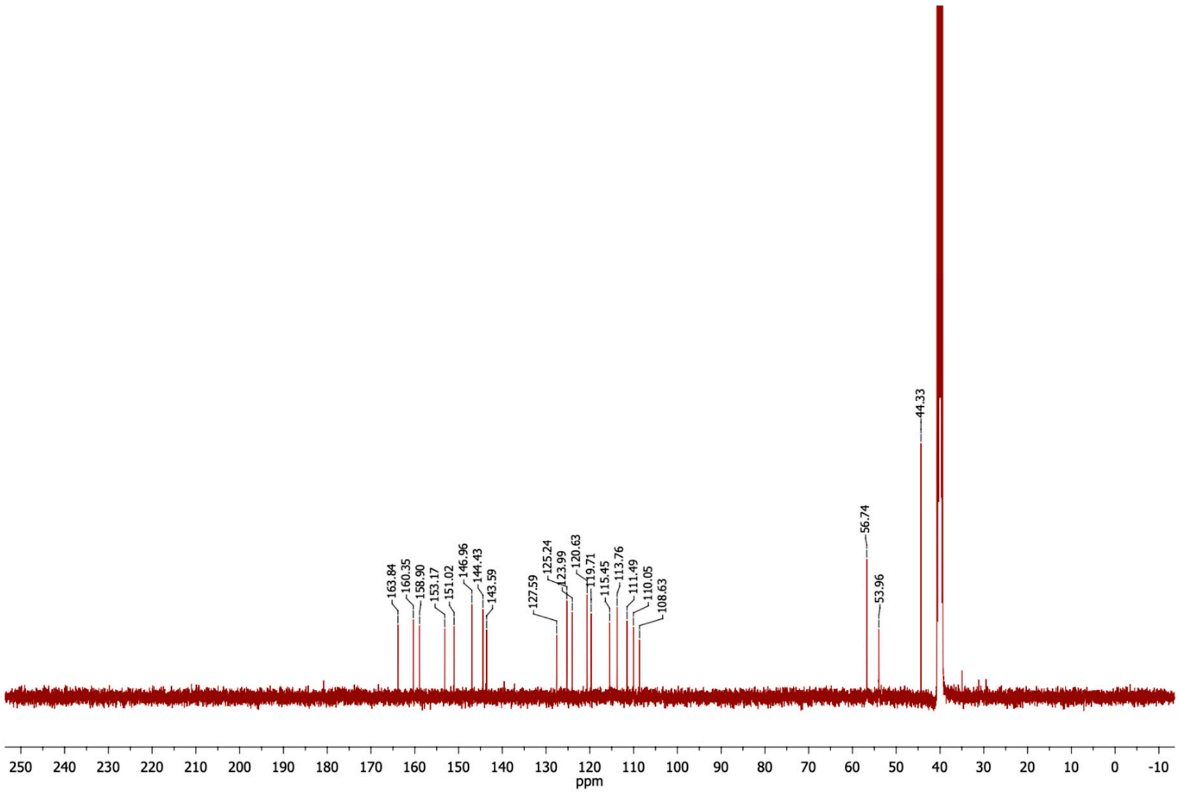

Figure S5. 8-((diethylamino)methyl)-4-(6-bromo-2-oxo-2*H*-chromen-3-yl)-7-hydroxy-2*H*-chromen-2-one (**3c**)

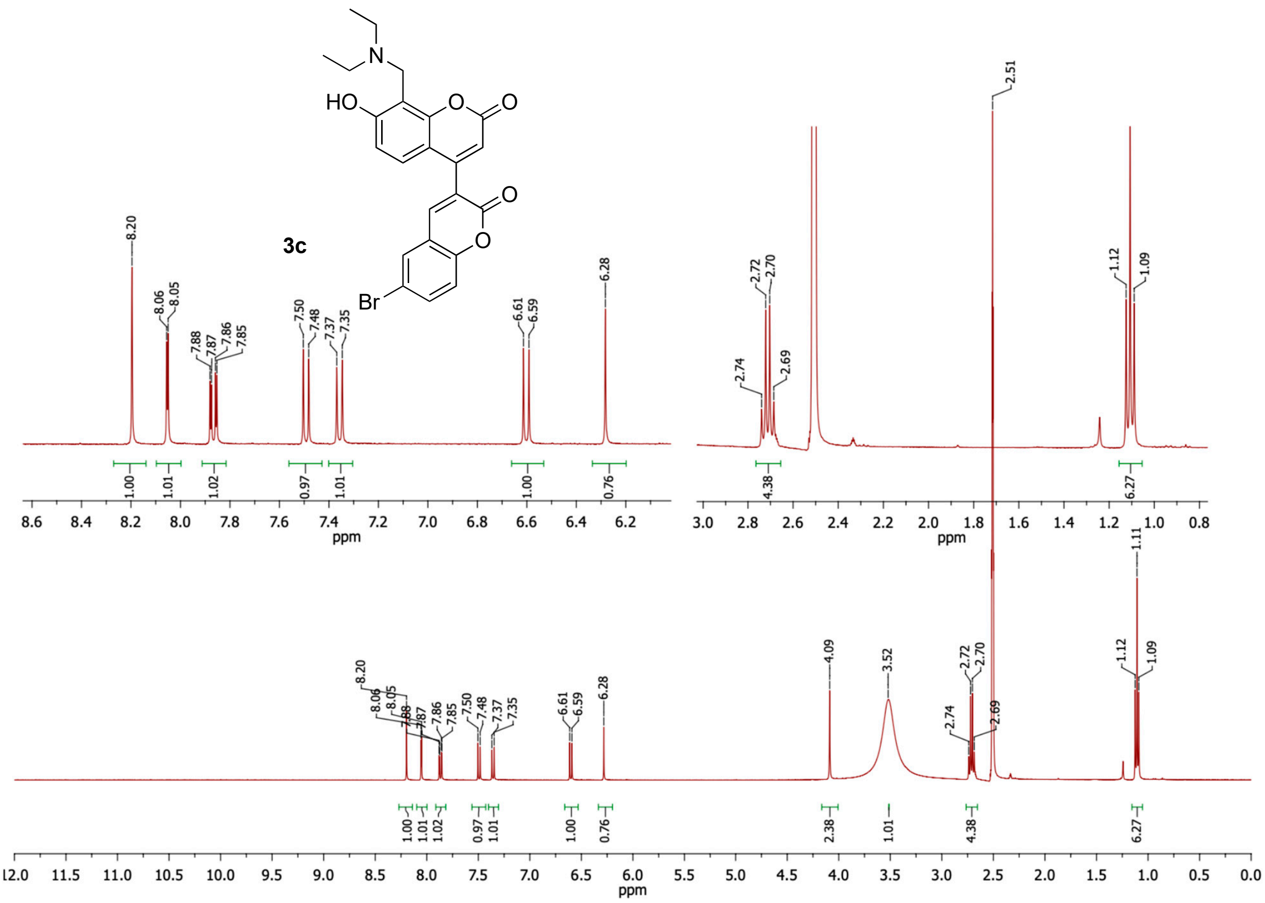

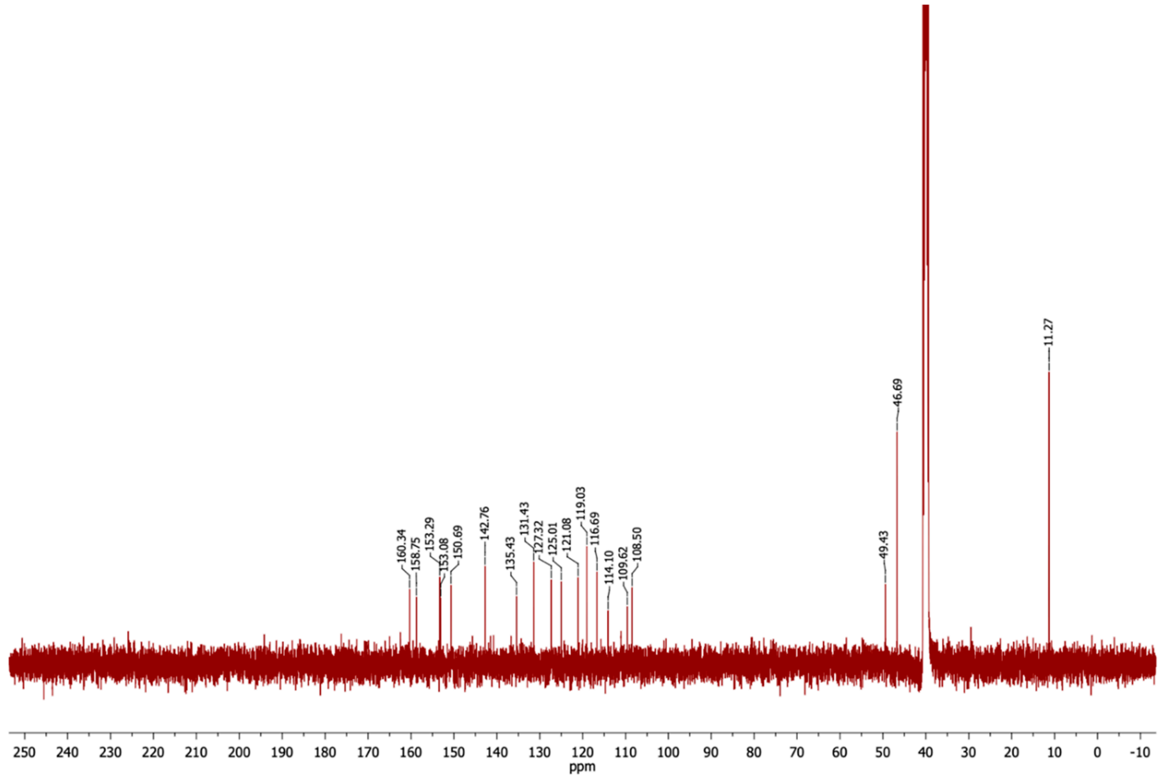

Figure S6. 8-((dipropylamino)methyl)-4-(8-methoxy-2-oxo-2*H*-chromen-3-yl)-7-hydroxy-2*H*-chromen-2-one (**4d**)

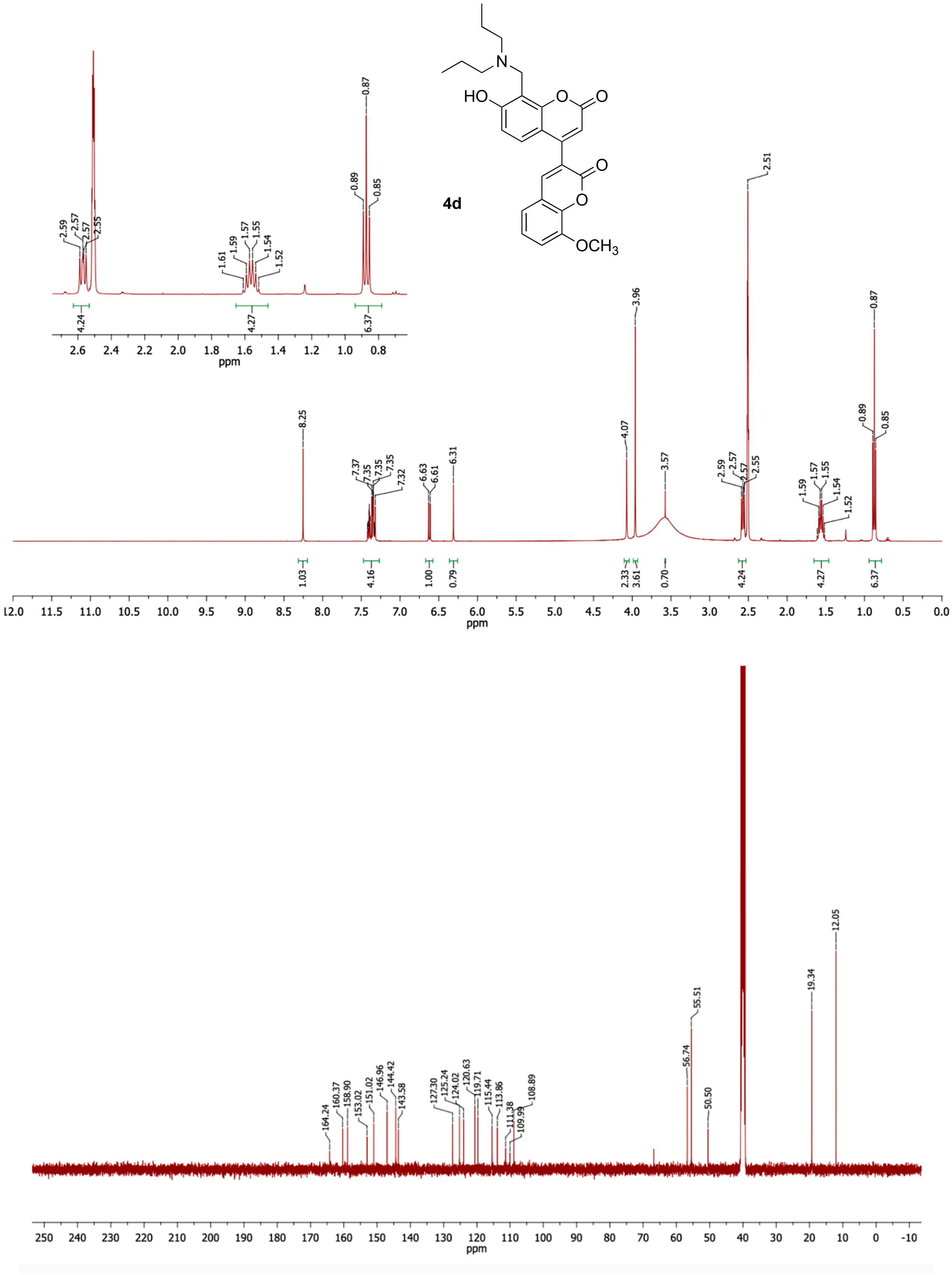

Figure S7. 8-((butyl(methyl)amino)methyl)-4-(8-methoxy-2-oxo-2*H*-chromen-3-yl)-7-hydroxy-2*H*-chromen-2-one (**5d**)

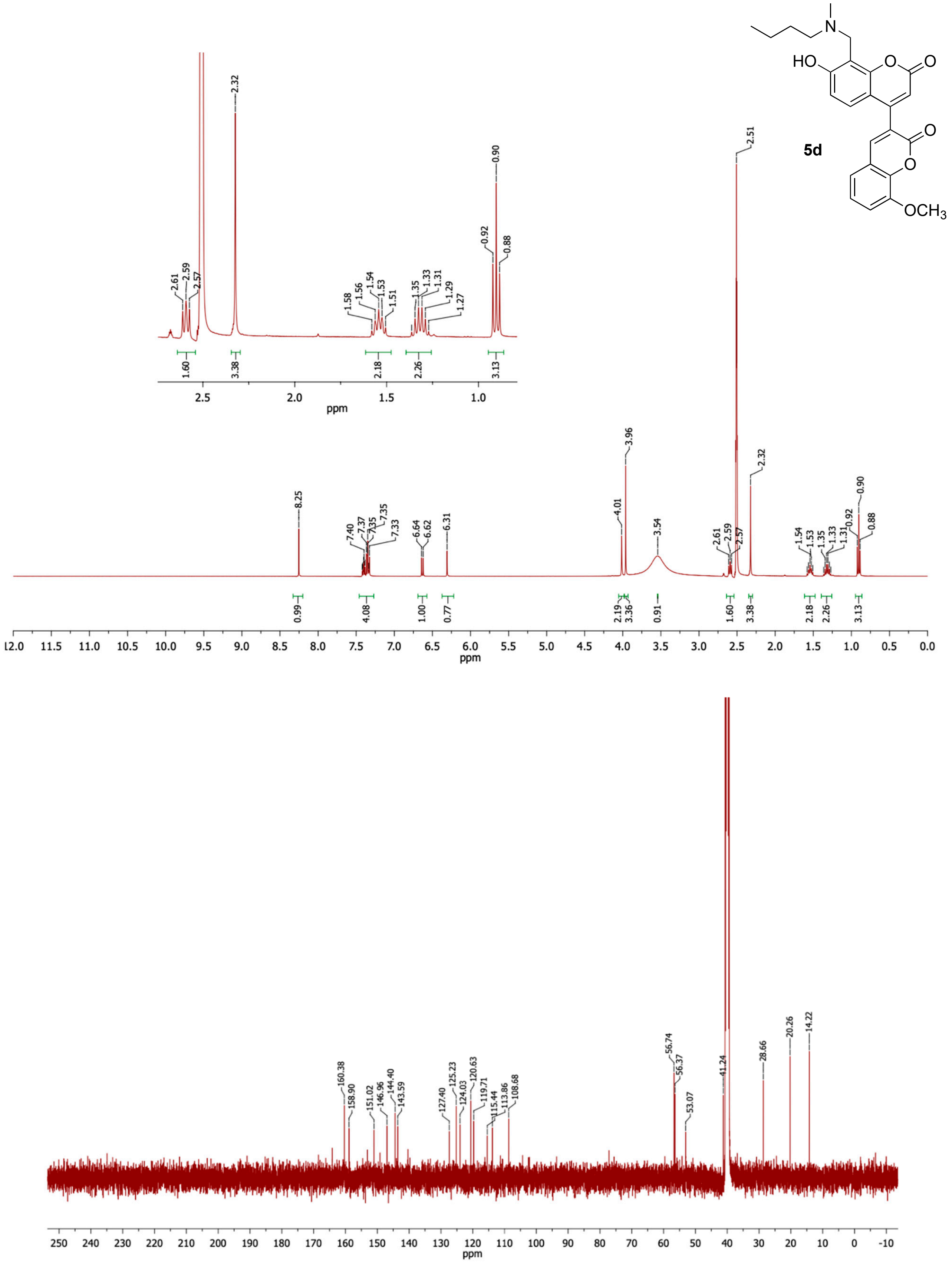

Figure S8. 8-((bis(2-methoxyethyl)amino)methyl)-4-(8-methoxy-2-oxo-2*H*-chromen-3-yl)-7-hydroxy-2*H*-chromen-2-one **(6d)**

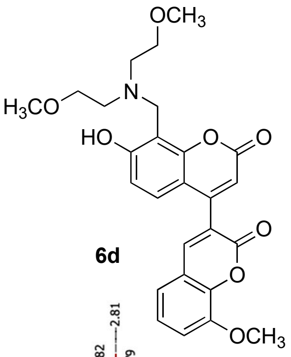

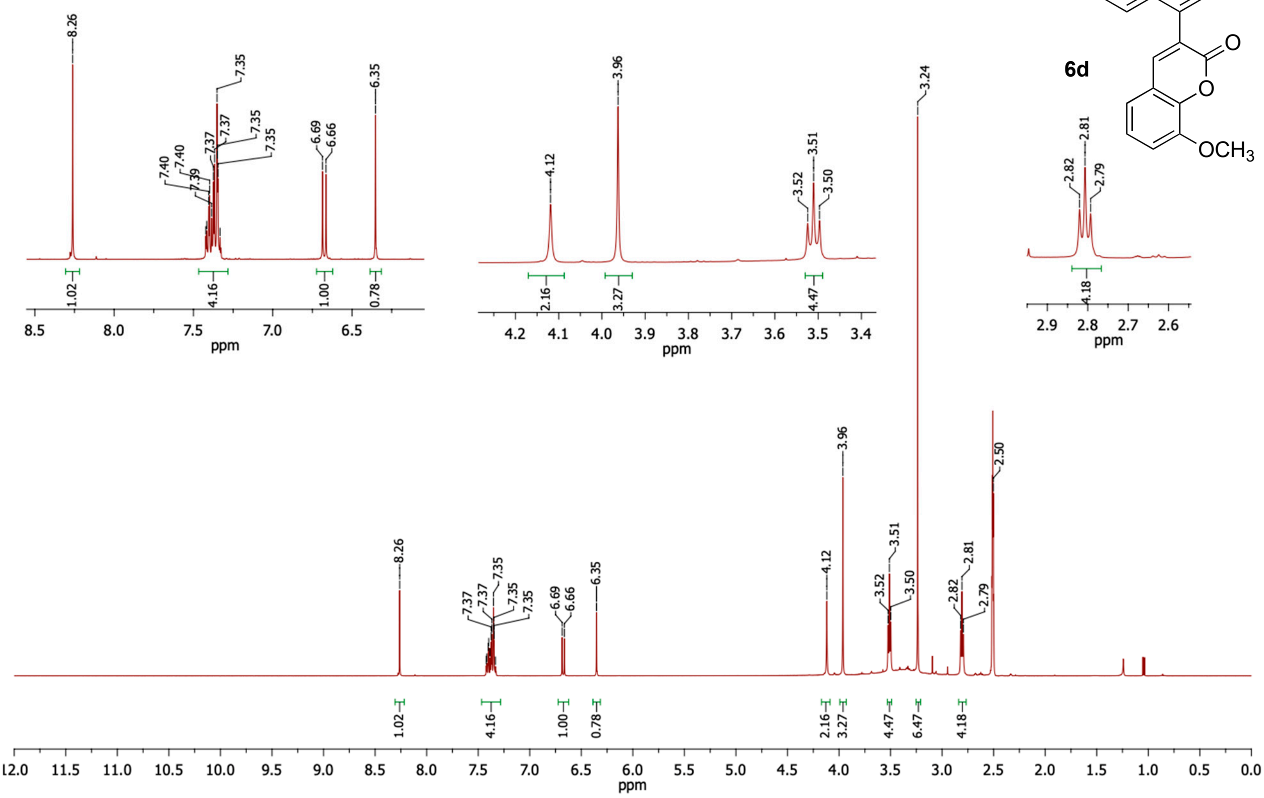

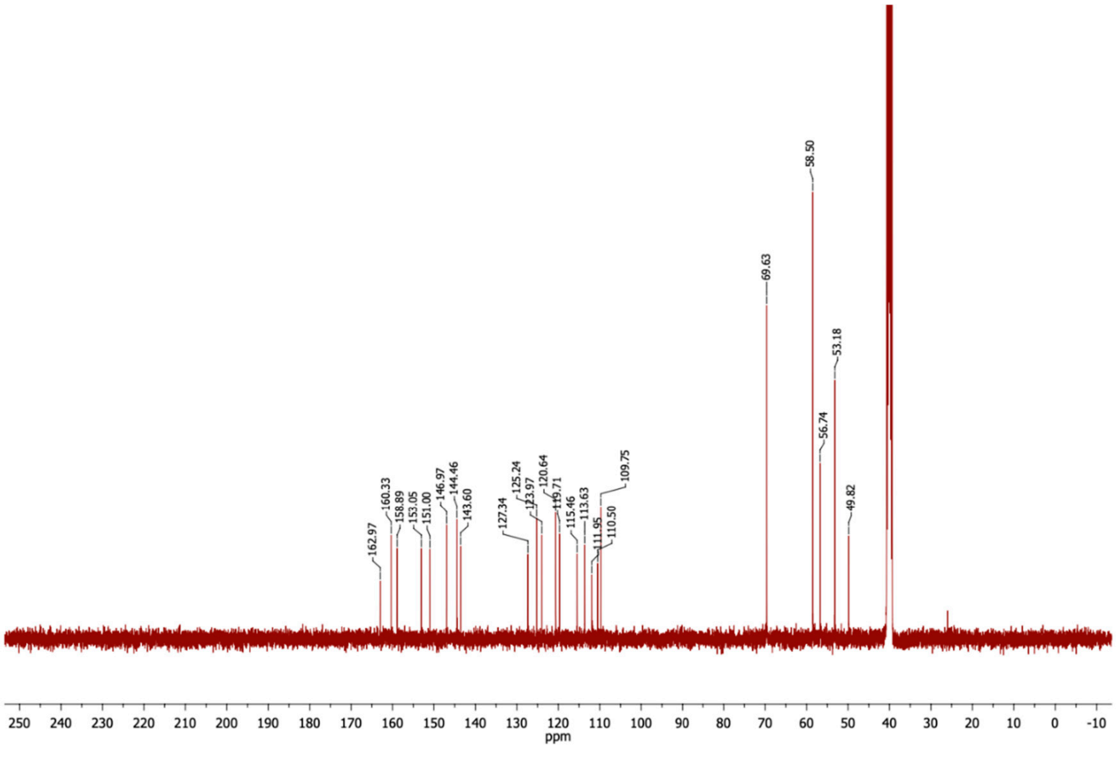

Figure S9. 8-(((benzyl(methyl)amino)methyl)-4-(8-methoxy-2-oxo-2*H*-chromen-3-yl)-7-hydroxy-2*H*-chromen-2-one (**8d**)

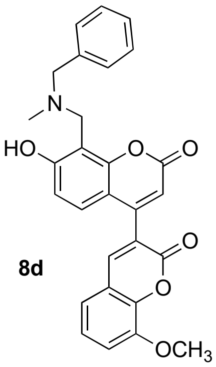

8d

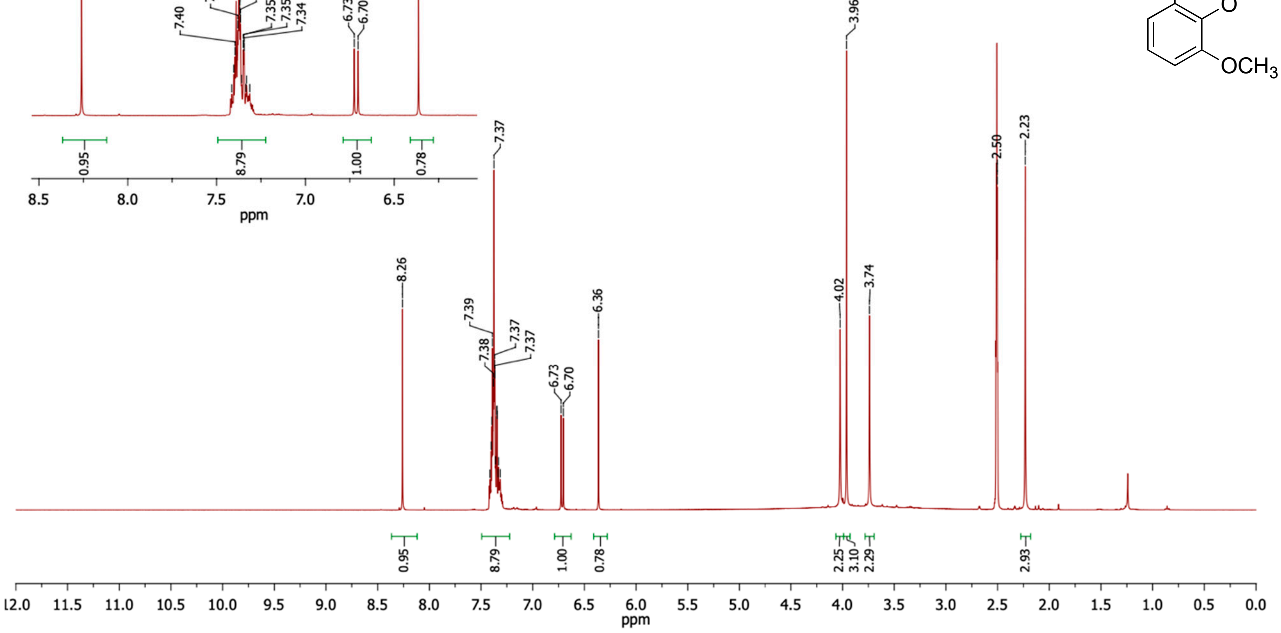

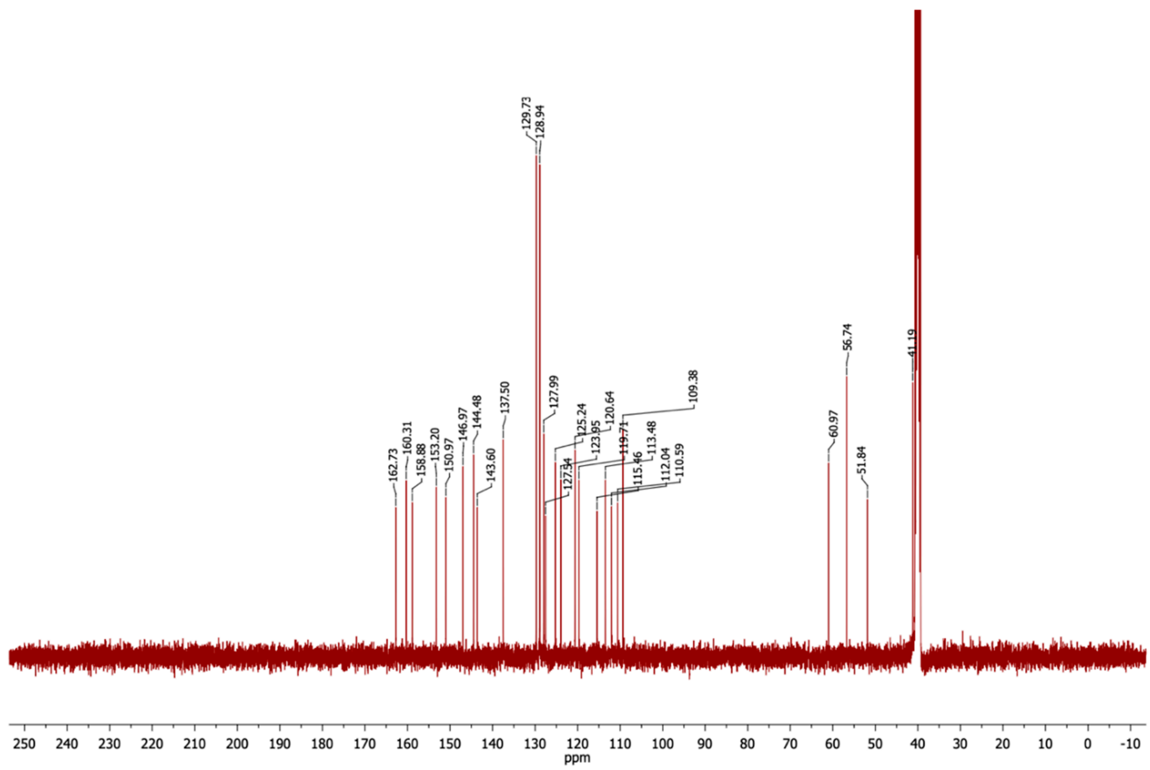

Figure S10. RP-HPLC chromatograms of the tested bis-coumarin derivatives

Chromatograms of bis-coumarin derivatives were recorded in the following conditions: mobile phase: MeOH/Water (acetic acid 0.5%) pH 2.8 at different percentages; stationary phase: Phenomenex, Kinetex 5 µ, C8, (150×3 mm); flux: 0.5 µL/min; injection: 2 µL; λ: 330 nm. Equipment: Agilent HPLC 1260 Infinity Series Integrated System (Agilent Technologies, Milan, Italy).

2a: MeOH/Water (acetic acid 0.5%) (20:80 v/v)

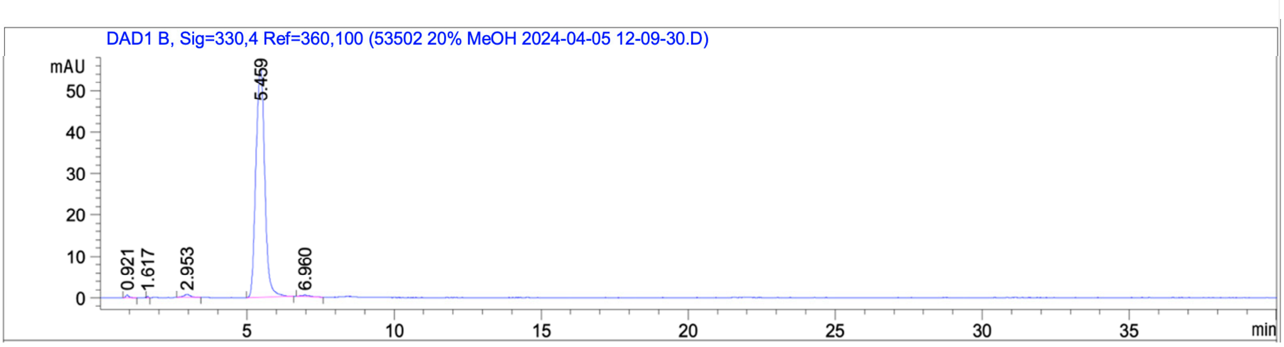

2b: MeOH/Water (acetic acid 0.5%) (30:70 v/v)

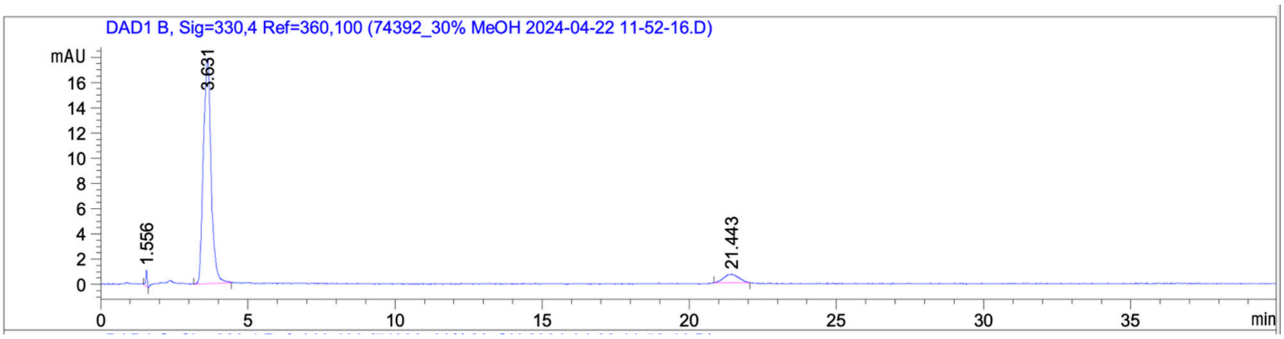

2c: MeOH/Water (acetic acid 0.5%) (30:70 v/v)

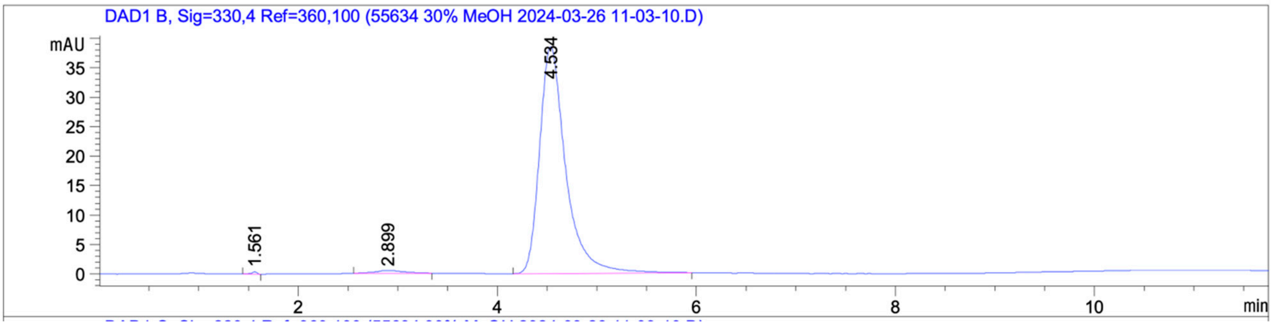

2d: MeOH/Water (acetic acid 0.5%) (20:80 v/v)

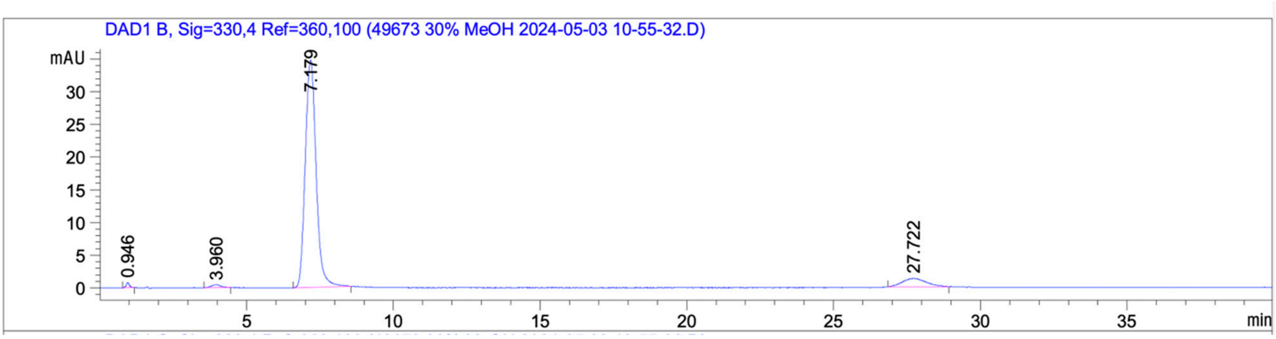

3c: MeOH/Water (acetic acid 0.5%) (30:70 v/v)

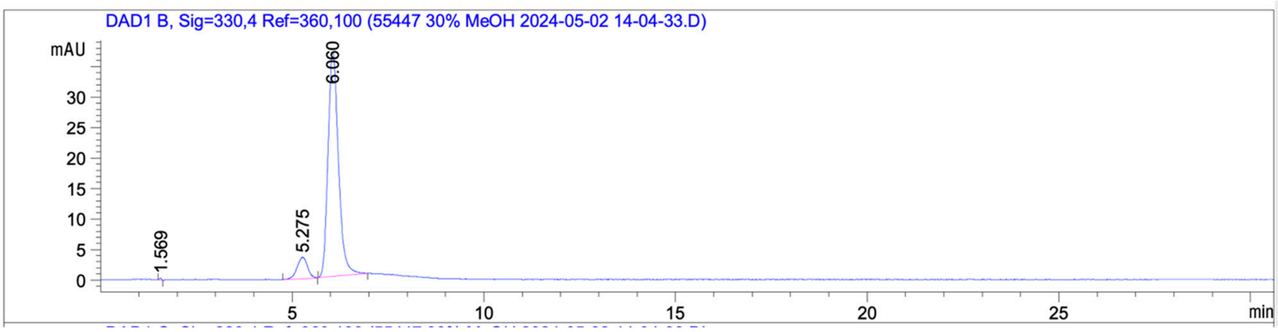

4a: MeOH/Water (acetic acid 0.5%) (30:70 v/v)

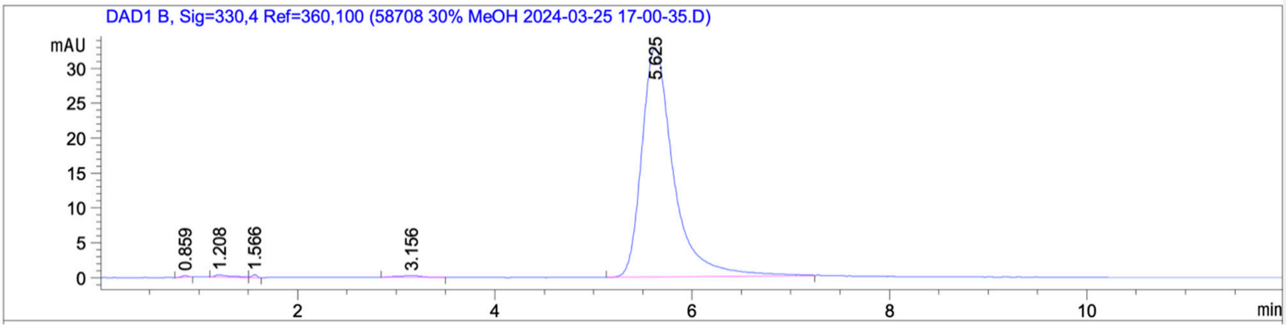

4d: MeOH/Water (acetic acid 0.5%) (30:70 v/v)

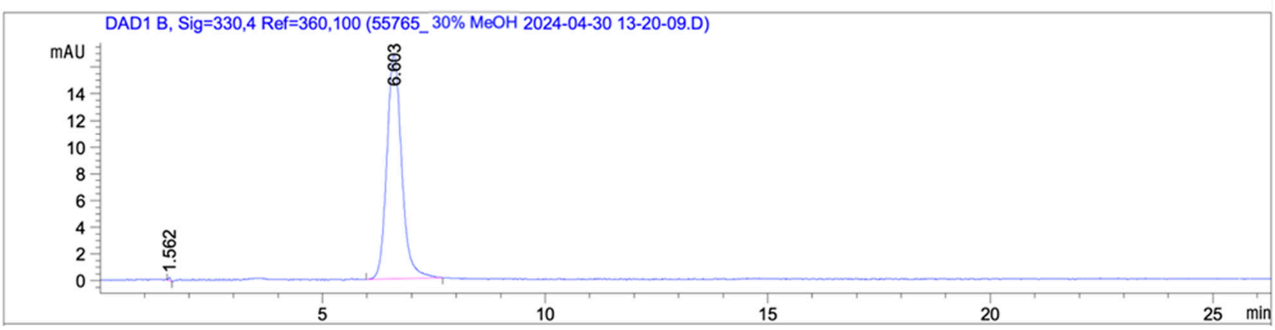

5a: MeOH/Water (acetic acid 0.5%) (30:70 v/v)

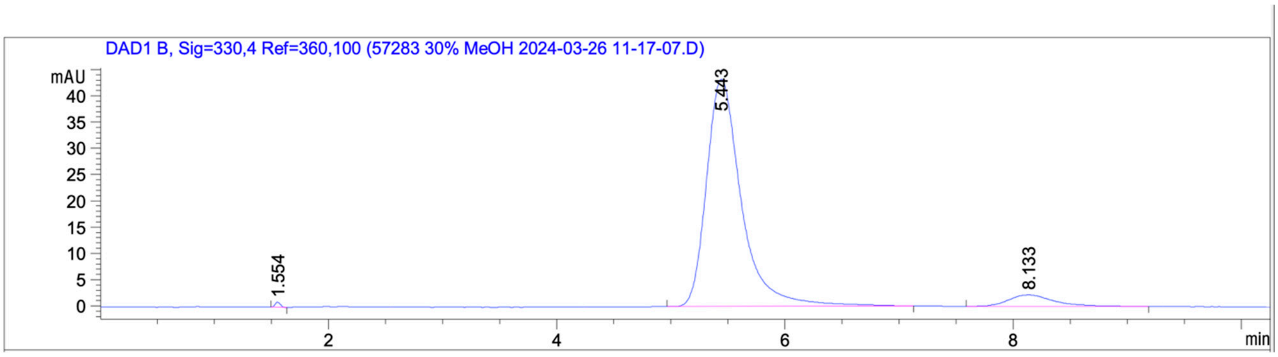

5b: MeOH/Water (acetic acid 0.5%) (35:65 v/v)

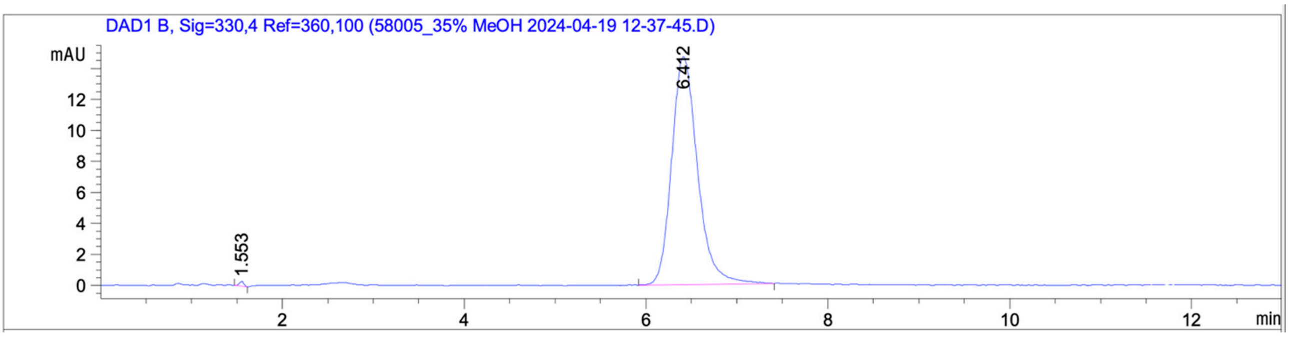

5c: MeOH/Water (acetic acid 0.5%) (35:65 v/v)

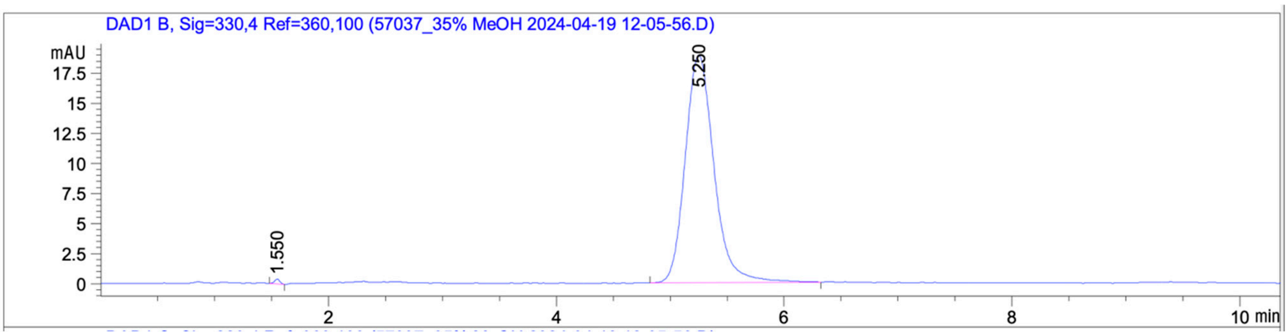

5d: MeOH/Water (acetic acid 0.5%) (30:70 v/v)

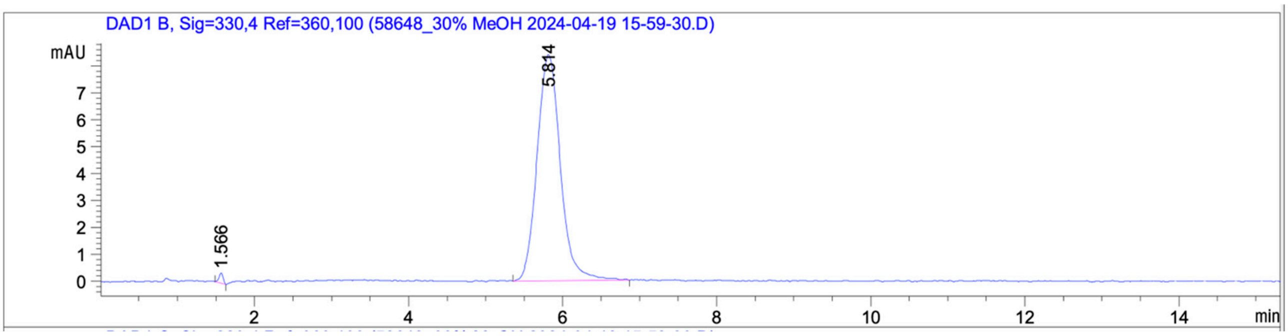

6a: MeOH/Water (acetic acid 0.5%) (30:70 v/v)

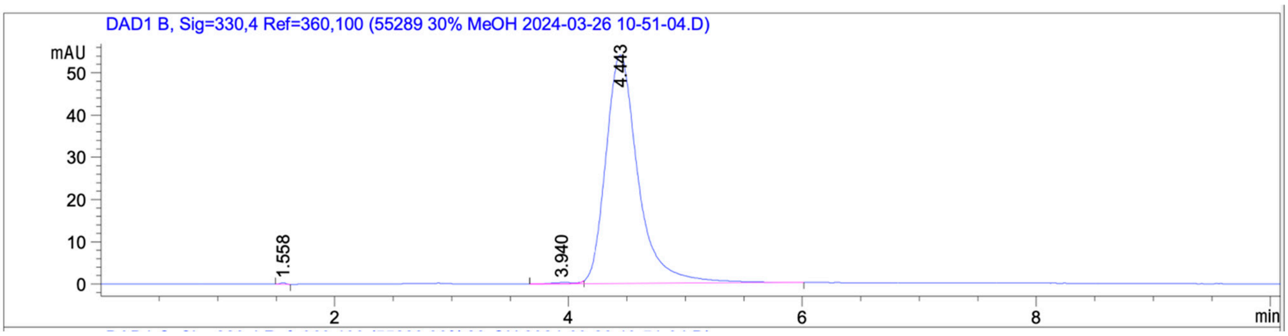

6b: MeOH/Water (acetic acid 0.5%) (35:65 v/v)

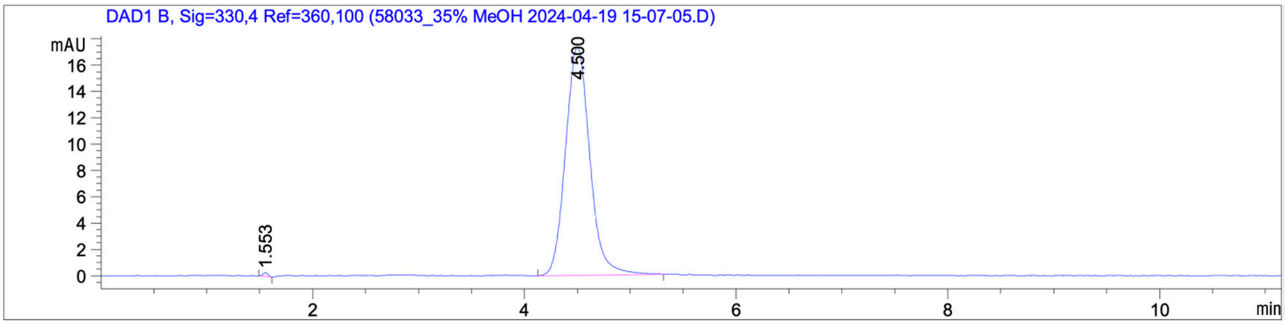

6d: MeOH/Water (acetic acid 0.5%) (30:70 v/v)

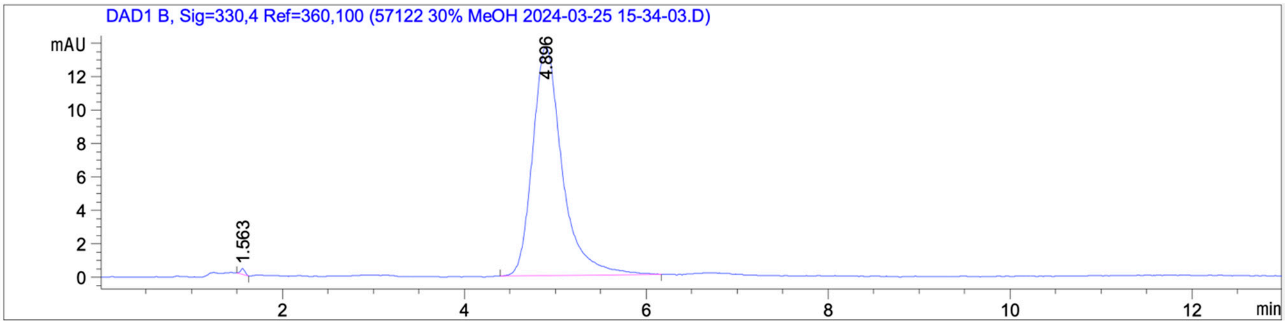

7a: MeOH/Water (acetic acid 0.5%) (40:60 v/v)

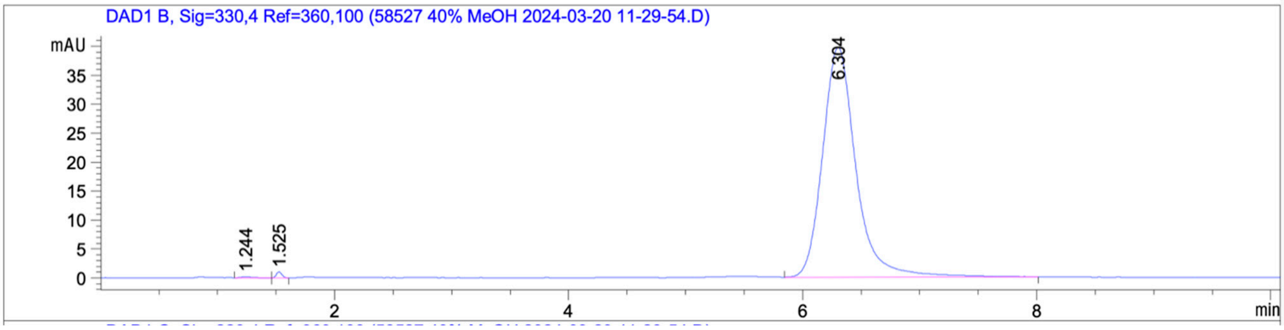

7d: MeOH/Water (acetic acid 0.5%) (40:60 v/v)

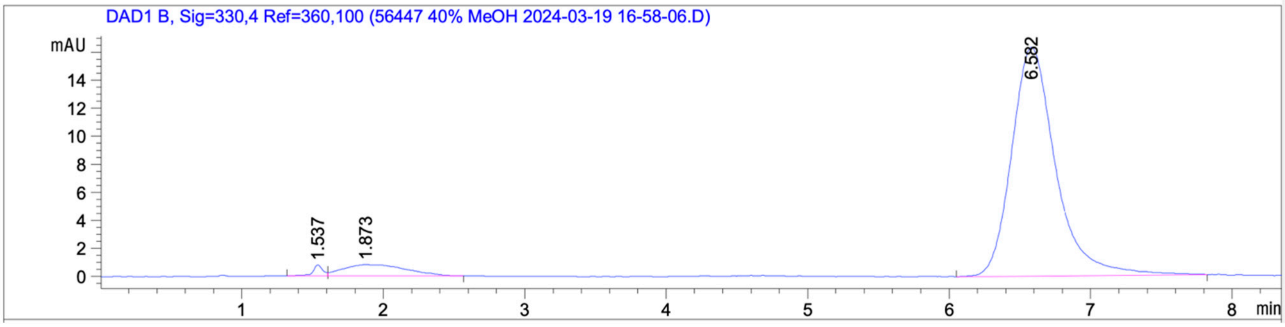

8a: MeOH/Water (acetic acid 0.5%) (30:70 v/v)

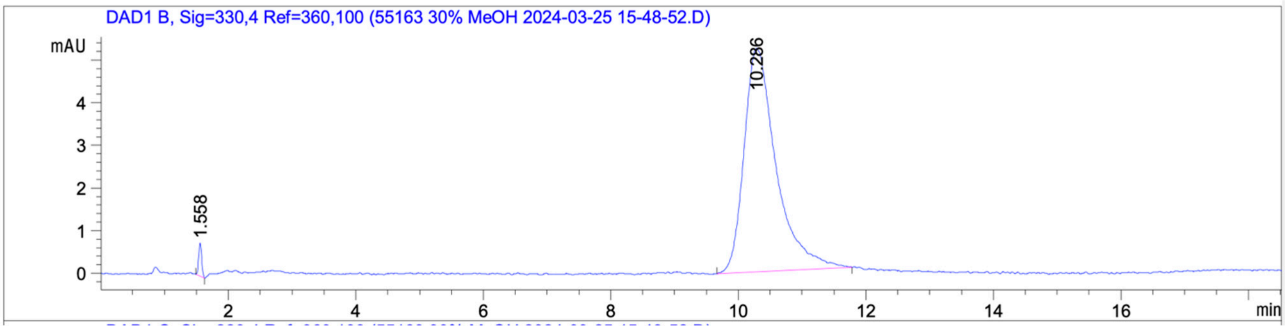

8b: MeOH/Water (acetic acid 0.5%) (35:65 v/v)

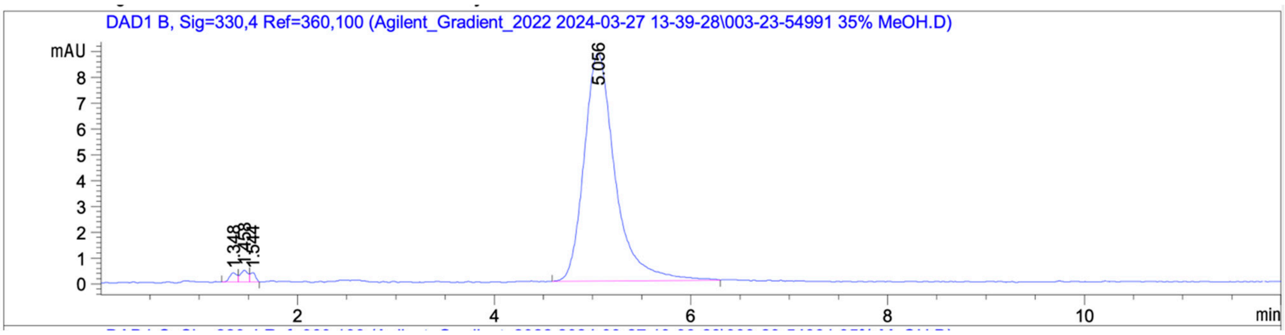

8c: MeOH/Water (acetic acid 0.5%) (35:65 v/v)

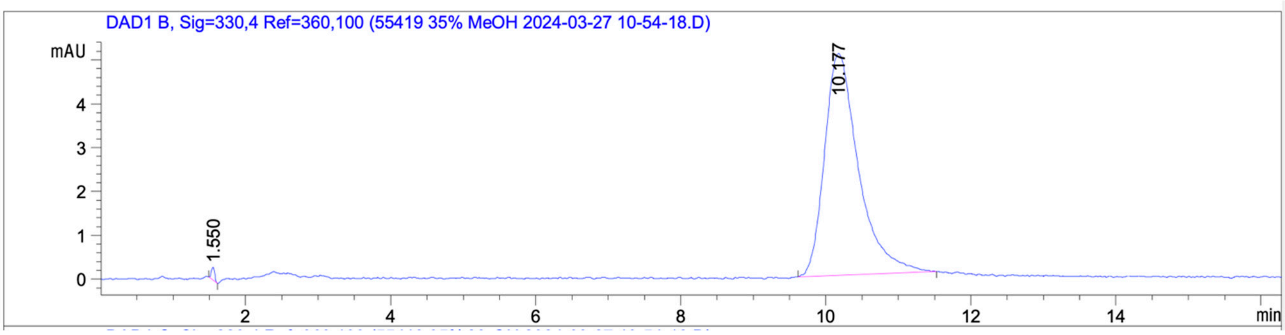

Figure S11. Time-dependent inhibition of human MAO-A by compounds **2b**, **5b** and clorgiline

Time courses of MAO-A inhibition by **2b** and **5b**, using a spectrophotometric assay measuring the absorbance of 4-hydroxyquinoline at 316 nm. The time traces of enzyme activity in the presence of inhibitors at a concentration close to IC_{50} (1 μM for **2b** and **5b**, 4 nM for clorgyline) are plotted. While the curves of test compounds are lowered compared with control, the curve of clorgyline, an irreversible inhibitor covalently binding to the enzyme, is completely flattened due to the enzyme inactivation.

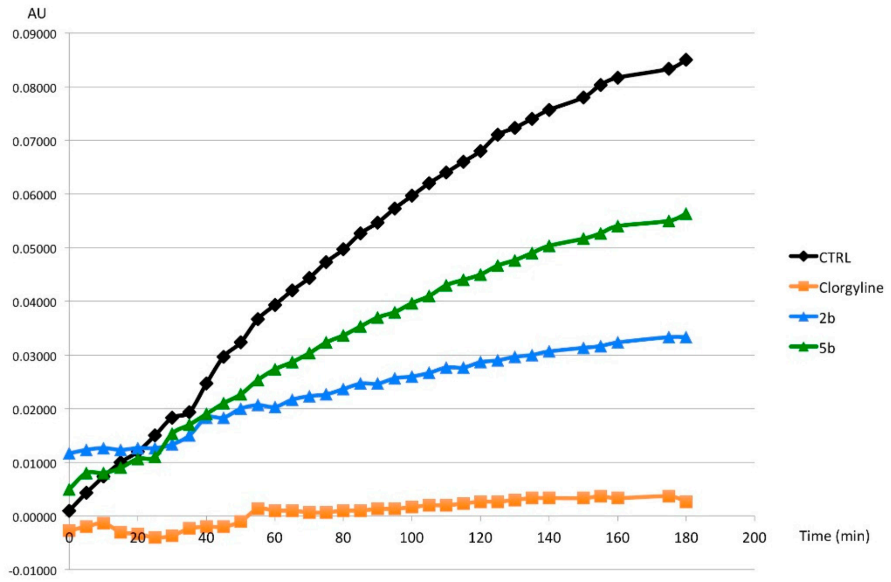

Table S1. Molecular docking results with MAOs A and B.

Cmpd	Free binding energy (kcal·mol-1) [a]		MAO A [b]		
	MAO A (2BXR)	MAO B (1GOS)	H-bonding	Hydrophobic interactions	Ionic interactions
**2a**	-11.38	-5.14	Ile207, Ser209, Tyr447	Phe208, Leu337, Tyr407, Tyr444	Glu216
**2b**	-11.25	-2.79	Ser209, Tyr444	Ala68, Tyr407, The352, Phe208, Tyr68, Met350, Ile180, Val93, Ile335	-
**2d**	-7.62	-3.26	Met445	Thr52, Tyr44	-
**4a**	-10.06	-1.28	Tyr69, Ser209, Tyr407	Phe208, Val303, Ile335, Phe352, Tyr444, Met445	Tyr407
**5a**	-7.82	-5.89	Ser209, Ile207	Leu97, Phe208, Leu337	-
**5b**	-10.98	-3.51	Ser209, Tyr444	Tyr68, Ala68, Ile180, Phe208, Met350, Ile335	-
**6a**	-6.55	-5.28	-	Phe208, Tyr407, Tyr444	-
**6d**	-5.17	-1.22	-	Phe208, Tyr444	-
**7a**	-5.03	-1.29	-	Phe208, Tyr444	-
**7d**	-6.52	-5.10	-	Phe208, Tyr407, Tyr444	-
**8a**	-7.23	-5.18	Ser209	Phe208, Leu337, Tyr407, Tyr444	-
**8d**	-4.56	-3.52	-	Val303, Ile325, Leu337	-
Moclobemide	-8.92	-2.57	Ser209, Tyr444	Leu97, Phe208, Ile325, Ile335, Leu337	-

[a] Free energy of binding (kcal·mol-1) estimated by AutoDock 4.2 software for ligands in the highest scored binding pose. [b] Residues in the binding site of MAO A mainly involved in the interaction with bis-coumarin ligands.

Table S2. Molecular docking results with acetylcholinesterase (AChE).

Comp.	Free binding energy (kcal·mol^{-1}) [a]	H-bonding [b]	Hydrophobic interactions [b]	Ionic interactions [b]
**2a**	-5.12	-	Phe331, Tyr334	-
**2c**	-2.13	-	-	-
**2d**	-3.59	-	-	-
**3c**	-9.15	Tyr70, Phe228	Phe330, Phe331, Tyr334, Leu282, Tyr279, Ile287, Phe290	-
**4a**	-4.56	-	Phe331, Leu282	-
**4d**	-7.15	Tyr70	Tyr279, Phe290	-
**5a**	-2.79	-	-	-
**5c**	-8.02	-	Trp84, Tyr279, Phe290	-
**5d**	-8.51	Trp84	Trp84, Tyr279, Ile287, Phe290	-
**6a**	-4.36	-	Phe330, Phe331	-
**6d**	-1.26	-	-	-
**7a**	-3.59	-	Tyr334	-
**7d**	-5.23	-	Trp84, Phe290	-
**8a**	-2.35	-	-	-
**8d**	-2.47	-	-	-
Donepezil	-9.53	-	Trp84, Ile144	Phe330, Trp84

[a] Free energy of binding (kcal·mol^{-1}) estimated by AutoDock 4.2 software for ligands in the highest scored binding pose. [b] Residues in the binding site of AChE mainly involved in the interaction with bis-coumarin ligands.

Table S3. SwissADME-assessed physicochemical properties, pharmacokinetics, drug-likeness and bioavailability scores of amphoteric bis-coumarin derivatives.

Property	Compounds								
	**2a**	**2b**	**2c**	**2d**	**3c**	**4d**	**5b**	**5c**	**5d**
**Molecular weight (g/mol)**	363.36	397.81	442.26	393.39	470.31	449.50	439.89	484.34	435.47
**N rotatable bonds**	3	3	3	4	5	8	6	6	7
**N H-bond acceptors**	6	6	6	7	6	7	6	6	7
**N H-bond donors**	1	1	1	1	1	1	1	1	1
**TPSA (Å^2)** [a]	83.89	83.89	83.89	93.12	83.89	93.12	83.89	83.89	93.12
**Consensus Log $P_{o/w}$** [b]	2.96	3.45	3.58	2.94	4.26	4.28	4.51	4.61	3.96
**Solubility (ESOL)** [c]	Moderate	Moderate	Moderate	Moderate	Moderate	Moderate	Moderate	Moderate	Moderate
**GI absorption**	High	High	High	High	High	High	High	High	High
**BBB permeant**	No	No	No	No	No	No	No	No	No
**P-gp substrate**	No	No	No	No	No	Yes	No	No	Yes
**Lipinski rule violations**	0	0	0	0	0	0	0	0	0
**Bioavailability Score** [d]	0.55	0.55	0.55	0.55	0.55	0.55	0.55	0.55	0.55
**PAINS (alert/s)** [e]	1	1	1	1	1	1	1	1	1

[a] TPSA: topological polar surface area (Å). [b] Arithmetic means of log $P_{o/w}$ calculation with five methods (iLOGP, XLOGP3, WLOGP, MLOGP, SILICOS-IT). [c] Water solubility calculated according to the ESOL method; accordingly, all the selected compounds are classified as 'moderately soluble', which means that $S < 1 \cdot 10^{-4}$ mol·L^{-1} (log $S < -4$). [d] Bioavailability score predicts the probability of a molecule having more than 10% oral bioavailability in rats; a value of 0.55 indicates that the compound can be effectively absorbed in the gastrointestinal tract after oral administration. [e] The only alert into the structure of the amphoteric bis-coumarins is for the Mannich base identified as one of the pan-assay interference compounds (PAINS).
